# Racialized economic segregation in relation to fecundability in a preconception cohort study

**DOI:** 10.1007/s11524-025-01038-y

**Published:** 2025-12-20

**Authors:** Sharonda M. Lovett, Erin J. Campbell, Andrea S. Richardson, Amelia K. Wesselink, Collette N. Ncube, Yvette C. Cozier, Lauren A. Wise, Mary D. Willis

**Affiliations:** 1 Department of Epidemiology, Boston University School of Public Health, Boston, MA USA; 2 Department of Behavioral and Policy Sciences, RAND Corporation, Pittsburgh, PA USA; 3 Department of Environmental and Occupational Health, George Washington University Milken Institute School of Public Health, Washington, DC USA

**Keywords:** Fertility, Fecundability, Structural racism, Socioeconomic factor, Neighborhood characteristics, Residential segregation, Index of Concentration at the Extremes

## Abstract

Racialized economic segregation is a known determinant of health disparities that has been associated with adverse pregnancy outcomes. However, no study has investigated its association with fecundability, the per-cycle probability of conception. We analyzed data from 10,438 U.S. participants enrolled in Pregnancy Study Online (2013–2022), an internet-based preconception cohort study. Eligible participants were aged 21–45 years, assigned female at birth, and trying to conceive without fertility treatment use. Using baseline residential addresses, we calculated segregation based on the Index of Concentration at the Extremes (ICE) from the American Community Survey 5-year estimates. ICE ranges from -1 (“disadvantaged”) to 1 (“privileged”). We operationalized ICE metrics at the census tract level: economic segregation (ICE_income_: ≥ $100 k vs. < $25 k), racial segregation (ICE_white/black_: non-Hispanic White vs. non-Hispanic Black), and racialized economic segregation (ICE_income + white/black_: non-Hispanic White ≥ $100 k vs. non-Hispanic Black < $25 k). We used proportional probabilities regression models to estimate fecundability ratios (FRs) and 95% confidence intervals (CIs), adjusting for precision variables. We observed a monotonic inverse association for increasing quintiles of ICE_income_ and ICE_income + white/black_ with fecundability, but less evidence of associations with ICE_white/black_. For instance, relative to ICE_income + white/black_ quintile 5 (“most privileged”), FRs for quintiles 4, 3, 2, and 1 (“most disadvantaged”) were 0.92 (95% CI = 0.86–0.99), 0.84 (95% CI = 0.78–0.90), 0.84 (95% CI = 0.78–0.90), and 0.79 (95% CI = 0.73–0.86), respectively. Living in the most disadvantaged neighborhoods with respect to racialized economic segregation was associated with a moderate decrease in fecundability.

**Supplementary Information:**

The online version contains supplementary material available at 10.1007/s11524-025-01038-y.

## Introduction

The co-occurrence of racial and economic segregation (hereafter “racialized economic segregation”) is a root cause of racial and socioeconomic disparities in health outcomes [1–3]. It is defined as the geographic separation of individuals with low income or from historically marginalized racial and ethnic groups into distinct residential environments owing to structural racism [1]. Deeply rooted in the history of slavery, black codes (*i.e.,* laws that governed the conduct of Black Americans), and Jim Crow laws (*i.e.,* the collection of statues that legalized racial segregation), racialized economic segregation manifests as a complex interplay of social, economic, and political forces. This phenomenon led to the concentration of Black Americans in less desirable neighborhoods facilitated by historical mortgage lending discrimination or “redlining” (defined as a historic racist practice that systematically diverted wealth away from Black and Hispanic neighborhoods and into White neighborhoods [4]) and racially driven zoning ordinances limiting land use. Although the Civil Rights Act of 1968 outlawed overt discrimination in the housing market [5, 6], contemporary racialized economic segregation persists in various forms such as racial steering (defined as the practice of guiding prospective home buyers or renters toward or away from certain neighborhoods based on their race or other characteristics), discriminatory lending practices (*e.g.,* net percentage of applications favoring privileged racial and ethnic groups) [7], and inequality in subsidized housing [8]. This confluence of racist practices, both codified and informal [9], is embodied in present-day health disparities.

**Fig. 1:**
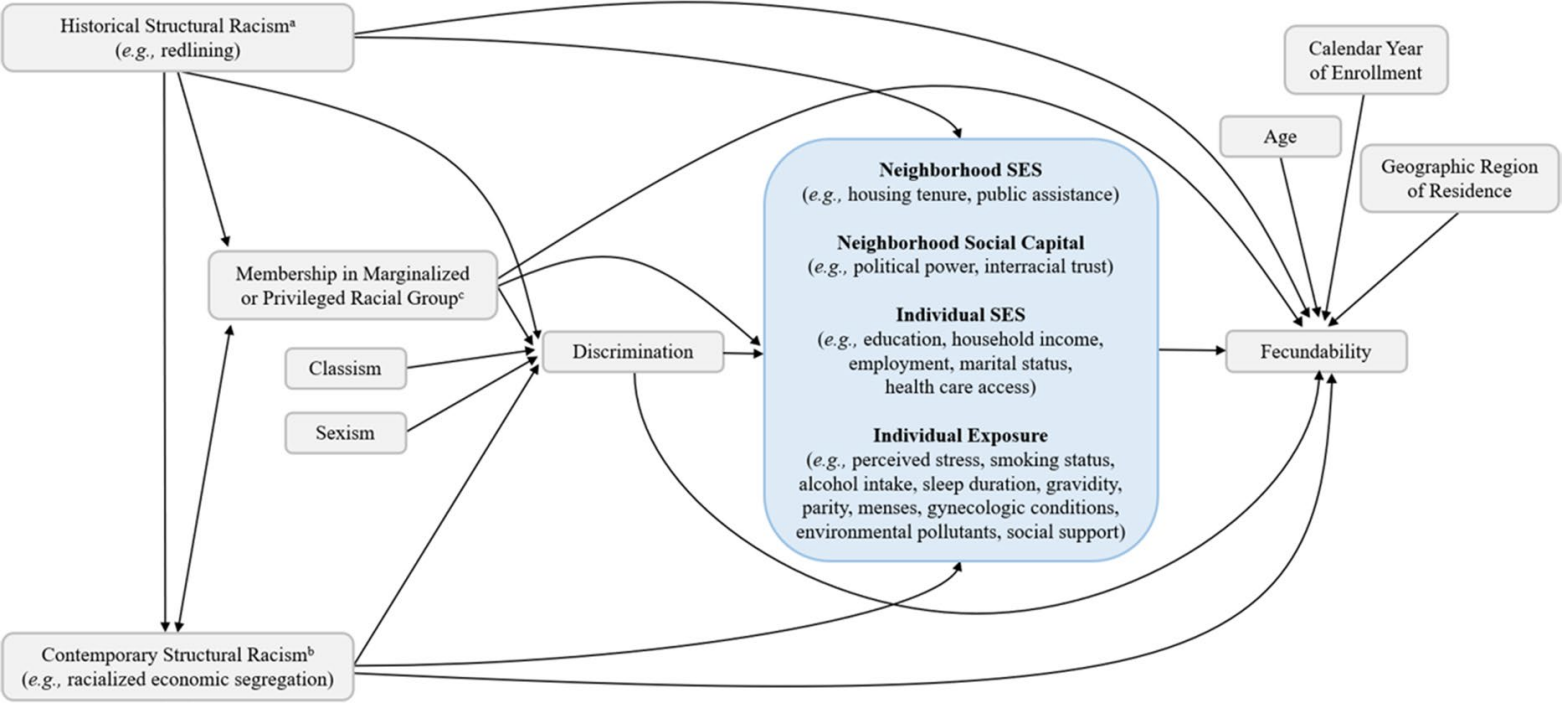
Simplified causal diagram of hypothesized pathways between racialized economic segregation and fecundability. Note: SES = socioeconomic status; This diagram was informed in part by Kramer, MR & Hogue, CR. Is segregation bad for your health? Epidemiol Rev. 2009; 31, 178–194. https://doi.org/10.1093/epirev/mxp001 and Howe CJ, Bailey ZD, Raifman JR, & Jackson JW. Recommendations for using causal diagrams to study racial health disparities. Am J Epidemiol. 2022; 191(12):1981–1989. https://doi.org/10.1093/aje/kwac140; ^a^Examples of historical structural and/or institutional racism include racist policies (*e.g.,* slavery, Jim Crow laws, redlining); ^b^Examples of contemporary structural and/or institutional racism (*e.g.,* segregation, discriminatory mortgage lending practices); ^c^ “Membership in marginalized or privileged racial group” was modeled in regression analyses using a variable on self-identified race/ethnicity

Residence in a segregated neighborhood has been found to increase risk of adverse pregnancy and birth outcomes, including hypertensive disorders of pregnancy [10, 11], shorter gestational age [12, 13], preterm birth [13–19], low birth weight [14, 16], and stillbirth [20]. No study to date, however, has investigated racialized economic segregation and fertility, an important reproductive health endpoint with substantial disparities across racial and socioeconomic groups [21–24]. We hypothesize segregation could become biologically embedded to influence fertility [25], largely through oxidative stress and systemic inflammation (Fig. 1) [26–28]. This hypothesis builds on existing literature that shows perceived stress and living in a socioeconomically disadvantaged neighborhood (defined using national and within-state ranks of material resources via the Area Deprivation Index) are associated with reduced fertility [29, 30]. Although neighborhood- and individual-level socioeconomic status are predictive of health, scholars agree that segregation is a dynamic spatial–temporal process of racial, ethnic, and socioeconomic stratification that begets conditions of neighborhood disadvantage and reduced opportunities of education and income [1, 2, 31]. In other words, segregation is a determinant of socioeconomic status that can independently, or jointly, increase exposure to psychosocial and environmental pollutants that link neighborhood or housing conditions to health.

Recent studies have shown that the built and social environment (*e.g.,* redlining [32, 33], green space [34], proximity to major roads [35], industrial facilities [36], air pollution [37], discrimination [38]) may also influence fertility outcomes. Given that 10–15% of reproductive-aged couples in the U.S. meet the clinical definition of infertility [21–23, 39], investigating structural drivers of neighborhoods that influence fertility is an important public health goal. Moreover, the structural and political elements that are present in neighborhood environments represent a complex issue encompassing reproductive, social, and environmental justice. Our central hypothesis is that segregation, conceptualized as a neighborhood-level stressor, is a potential driver of racial and socioeconomic disparities observed across the fertility spectrum, including those who conceive quickly or take ≥ 12 months to conceive [40–42].

To address this gap in the literature, we used data from a preconception cohort study to evaluate associations between economic, racial, and racialized economic segregation with fecundability (*i.e.,* the per-cycle probability of conception [43]).

## Methods

### Study Population

Pregnancy Study Online (PRESTO) is an internet-based preconception cohort study of pregnancy planners residing in the U.S. and Canada [44]. Participants are recruited through social media advertisements (*e.g.,* Facebook, Instagram) and flyers in medical centers. Eligible participants were assigned female at birth, aged 21–45 years, and attempting spontaneous conception without fertility treatment use. Participants completed a baseline questionnaire and follow-up questionnaires every 8 weeks for up to 12 months or until reported pregnancy, whichever occurred first.

PRESTO was approved by the Institutional Review Board at Boston University Medical Campus. All participants provided informed consent.

### Exposure Assessment

Using 5-year estimates from the American Community Survey, which was obtained through the National Historical Geographic Information System [45, 46], we extracted sociodemographic data for all U.S. census tracts that corresponded to participants’ calendar year of enrollment (2013–2022). We calculated census tract metrics for the Index of Concentration at the Extremes (ICE) for all U.S. census tracts, before subsetting to census tracts that aligned with PRESTO participants’ geocoded residential address at baseline (eTable 1). ICE is a validated metric of spatial polarization quantifying neighborhoods along the “continuum of concentrated affluence and poverty” [47]. This metric is calculated as: ICE_i_ = (A_i_-P_i_)/T_i_, where A_i_ = number of “privileged” persons, P_i_ = number of “disadvantaged” persons, and T_i_ = total population in the census tract. ICE ranges from −1.0 (*i.e.,* participants reside in neighborhoods where all residents are “disadvantaged”) to 1.0 (*i.e.,* participants reside in neighborhoods where all residents are “privileged”), showing directionality of concentrated extremes [48]. A value of 0 theoretically indicates the neighborhood is integrated (*i.e.,* equal numbers of disadvantaged and privileged persons), which is often implausible given U.S. patterns of segregation [48]. ICE metrics perform best when using census tracts as the spatial unit vs. city/town [49]. For example, economic segregation (ICE_income_) represented as ≥ $100 k vs. < $25 k, racial segregation (ICE_white/black_) represented as non-Hispanic White vs. non-Hispanic Black, and racialized economic segregation (ICE_income + white/black_) represented as non-Hispanic White ≥ $100 k vs. non-Hispanic Black < $25 k within a given census tract. For joint assessment of race/ethnicity and income, we calculated an expanded version of ICE (eTable 1) [48, 50, 51], including racialized economic segregation represented as non-Hispanic White ≥ $100 k vs. Hispanic < $25 k (ICE_income + white/hispanic_) and non-Hispanic White ≥ $100 k vs. Asian < $25 k (ICE_income + white/asian_). We categorized each ICE metric into quintiles based on distributions in the cohort (eTable 2).

### Outcome Assessment

We calculated time-to-pregnancy (TTP) using menstrual data from baseline and follow-up questionnaires [44]. On the baseline questionnaire, participants reported the date of their last menstrual period (LMP) and whether their menstrual cycles were regular. Participants with regular cycles reported their typical cycle length. Participants with irregular cycles reported the number of menses in a year and the estimated number of days until their next menses. On follow-up questionnaires, participants reported their LMP date, current pregnancy status, and if they had initiated fertility treatment since their last questionnaire [44]. Among participants who conceived, we asked how their pregnancy was confirmed (*e.g.,* home pregnancy test). We calculated TTP in discrete menstrual cycles: (cycles of attempt at study entry) + [(LMP date from most recent follow-up questionnaire − date of baseline questionnaire completion)/usual cycle length] + 1.

### Mediator Assessment

We hypothesized there were several potential mediators in the association between economic, racial, and racialized economic segregation with fecundability. At baseline, we asked participants about cycle regularity (“has your menstrual period been regular on its own without the use of hormonal contraceptives”), menstrual cycle length, educational attainment, current unemployment status, current body mass index (BMI; with ≥ 30 kg/m^2^ representing high BMI), current smoking status, current alcohol intake, and sleep duration [hours/night]. Participants also provided data on reproductive history (sexually transmitted infections [STI; defined as chlamydia, genital herpes, or genital warts], uterine leiomyomata, endometriosis, polycystic ovary syndrome) and mental health (current perceived stress via the 10-item version of the Perceived Stress Scale [PSS; range: 0–40, with a score ≥ 25 indicating high perceived stress] [52] and current depressive symptoms via the 12-item Major Depression Inventory [MDI; range: 0–50, with a score ≥ 30 classified as severe depressive symptoms]) [53, 54].

### Other Covariates

Participants provided individual-level data on the baseline questionnaire: race, ethnicity, highest level of parental educational attainment (mother or father), household income, age at menarche, gravidity, parity, multivitamin use, last method of contraception, factors related to intensity of trying to conceive (intercourse frequency, doing something to improve chances of conception [*e.g.,* charting menses]), infertility history before enrollment (*i.e.,* tried to conceive for ≥ 12 months without success), and frequency of visits to a primary care provider in the past year.

### Missing Data

We used fully conditional specification methods to multiply impute missing covariate and outcome data [55, 56]. Missingness ranged from < 0.1% (age) to 6.2% (parental educational attainment). For participants who did not complete any follow-up questionnaires (12.8%), we assigned one menstrual cycle of follow-up and imputed their pregnancy status at the end of that cycle (yes vs. no). We generated 20 imputation datasets across which we combined coefficient and standard error estimates [57].

### Exclusions

Between June 2013 and December 2022, 16,912 eligible participants completed the baseline questionnaire (**eFigure 1**). We excluded participants who reported residential addresses in Canada (n = 2,705, due to transnational differences in segregation), reported residential addresses outside of the conterminous U.S. (*e.g.,* Hawaii and Alaska, n = 109), and reported residential addresses that could not be accurately geocoded (n = 9). We also excluded participants with delayed completion of the baseline questionnaire (n = 43); whose LMP was > 6 months before completing the baseline questionnaire, unknown, or implausible (n = 133); and who did not experience menses during follow-up (n = 30). We then excluded participants who had been attempting pregnancy for > 6 cycles at study entry (n = 2,843, to reduce potential for reverse causation bias [58]). In the main analysis, we additionally excluded 602 participants with a zip-code-level geocode of residential address (*i.e.,* lower quality spatial geocodes). The final analytic sample comprised 10,438 U.S. female participants with a street-level geocode.

### Statistical Analysis

Participants contributed observed menstrual cycles from study entry until pregnancy or a censoring event (*e.g.,* loss to follow-up, 12 cycles of attempted conception), whichever came first. We applied life-table methods to compute the cumulative percentage of participants who conceived during follow-up, accounting for censoring events [59]. We implemented an Andersen-Gill data structure [60, 61] to account for left truncation from delayed entry into the risk set [62, 63]. We used proportional probabilities regression models [64] to estimate fecundability ratios (FRs) and 95% confidence intervals (CIs), comparing each category of exposure with the reference group (*i.e.,* highest quintile of each ICE metric [“most privileged”]). A FR < 1 signifies reduced fecundability or longer TTP. We fit restricted cubic splines to examine the potential for non-linear associations [65, 66].

We selected potential confounders and precision variables based on a priori literature and an assessment of a causal diagram (Fig. 1) [2, 67]. The first set of multivariable models adjusted for age (< 25, 25–29, 30–34, ≥ 35 years), calendar year of enrollment (2013–2022), and geographic region of residence (U.S. Northeast, South, Midwest, West) as precision variables. The second set additionally adjusted for a wider set of precision variables (*i.e.,* characteristics with a strong relationship with the outcome): multivitamin use (yes vs. no), last method of contraception (oral contraceptives, other hormonal methods, barrier methods, withdrawal/other), and intercourse frequency (< 1, 1, 2–3, ≥ 4 times/week). The third set additionally adjusted for modern sociodemographic groups: participants’ race/ethnicity (non-Hispanic White; non-Hispanic Black; Hispanic; non-Hispanic Asian, Native Hawaiian, or Pacific Islander; non-Hispanic multiracial; non-Hispanic some other race) [68], educational attainment (≤ 12, 13–15, 16, ≥ 17 years), and household income (< $50,000, $50,000-$99,999, $100,000-$149,999, ≥ $150,000). Because individual-level race/ethnicity, socioeconomic variables, and clinical-related factors could be an upstream or downstream effect of neighborhood environments [14, 69], we argue these covariates are likely mediators of the segregation-fecundability association; thus, our a priori preferred model for interpretation is the first set.

We also examined the extent to which associations between racialized economic segregation and fecundability were mediated through individual-level factors at baseline. In exploratory mediation analyses with exposure-mediator interaction [70], we estimated the natural indirect effects of each mediator on the natural direct effect of racialized economic segregation (ICE_income + white/black_: quintile 1 vs. 5) and fecundability. We calculated the percentage mediated as: (FR_NDE_ × [FR_NIE_ – 1])/(FR_NDE_ × FR_NIE_ – 1).

### Sensitivity Analysis

We stratified analyses by pregnancy attempt time at enrollment (< 3 vs. 3–6 cycles), parity (0 vs. ≥ 1 births), and infertility history (yes vs. no). We also restricted analyses to nulligravid participants with < 3 cycles of pregnancy attempt time to assess the extent to which selection bias may have influenced our results. For example, couples may relocate after having children [71–73] and couples with a history of infertility may be more likely to enroll in our study. We additionally stratified by BMI (< 25, 25–29, ≥ 30 kg/m^2^), race/ethnicity (non-Hispanic White; non-Hispanic Other Race [defined as Black; Asian, Native Hawaiian, or Pacific Islander; American Indian, Alaskan Native, or Indigenous; Middle Eastern or North African; multiracial; some other race; missing race], Hispanic), educational attainment (≤ 12, 13–15, 16, ≥ 17 years), and household income (< $50,000, $50,000-$99,999, $100,000-$149,999, ≥ $150,000) to evaluate their potential to serve as modifiers of the segregation-fecundability association. Due to sparse data, we were only able to stratify by broad groupings of race/ethnicity.

In secondary models, we 1) restricted to participants who resided in the same zip-code within the 12 months prior to enrollment (*i.e.,* same, or similar neighborhood exposures) and 2) expanded the analytic sample to include participants with zip-code-level geocodes.

### Statistical Software

All analyses and visualizations were conducted in SAS version 9.4 and R version 4.4.0.

## Results

We followed 10,438 participants for 42,575 menstrual cycles, corresponding to 6,238 pregnancies. During 12 cycles of attempt time, 73.8% of participants conceived after accounting for censoring using life-table methods. Fourteen percent of participants attempted pregnancy for 12 cycles without conception, 7.3% initiated fertility treatment, 2.4% stopped trying to conceive, and 18.1% were loss to follow-up. Participants who were and were not lost to follow-up were similar according to mean age (30.0 vs. 30.3 years), but differed on several characteristics, such as infertility history (19.1% vs. 6.6%; eTable 3).

Mean baseline age was 30.2 (standard deviation [SD]: 4.1 years; Table 1). The majority of participants identified as non-Hispanic White (83.4%), attained ≥ 17 years education (42.9%), and < $100,000 in household income (53.6%). Participants’ residences spanned all 48 states in the conterminous U.S. (eFigure 2), predominantly in urban areas (95%; Table 1). Participants living in the most disadvantaged neighborhoods with respect to racialized economic segregation reported lower educational attainment and household income compared to participants living in the most privileged neighborhoods (ICE_income + white/black:_ quintile 1 vs. 5; Table 1). For economic segregation (ICE_income_) and racial segregation (ICE_white/black_), we found similar patterns of increasing degrees of sociodemographic disadvantage in disadvantaged quintiles (eTable 4). Non-Hispanic Black participants were most likely to reside in neighborhoods with greater extreme concentrations of education (ICE_education_), race (ICE_white/black_), and income + race (ICE_income + white/black_), while participants who identified as non-Hispanic Asian, Native Hawaiian, or Pacific Islander were more likely to reside in neighborhoods with lower economic segregation (“privileged”) (eTable 5). Spearman correlation coefficients ranged from 0.04 (ICE_education_ vs. ICE_white/asian_) to 0.98 (ICE_income + white/black_ vs. ICE_income + white/asian_; eTable 6).

**Table 1:** Demographic characteristics, overall and stratified by racialized economic segregation, PRESTO 2013–2022

Characteristic^b^	Overall	Racialized economic segregation (ICE_income + white/black_ score)^a^				
		Q1 (most disadvantaged)	Q2	Q3	Q4	Q5 (most privileged)
	(*n* = 10,438)	(*n* = 1,851)	(*n* = 2,011)	(*n* = 2,097)	(*n* = 2,205)	(*n* = 2,274)
Age (years), mean	30.2	29.7	29.6	30.1	30.5	31.0
Married, %	89.8	84.0	87.4	89.7	92.3	93.8
Residence in an urban area, %	95.0	97.2	92.2	92.4	95.3	97.7
Geographic region of residence, %						
U.S. Northeast	26.4	16.8	18.7	23.5	30.1	39.2
U.S. South	27.8	42.7	32.2	24.7	22.8	19.8
U.S. Midwest	26.5	24.1	26.8	29.6	27.2	24.7
U.S. West	19.4	16.4	22.2	22.2	20.0	16.2
Race/ethnicity, %						
Non-Hispanic White	83.4	71.9	84.2	85.4	85.6	88.2
Non-Hispanic Black	3.3	10.6	2.9	2.2	1.3	0.9
Hispanic	7.4	10.8	7.2	6.5	6.7	6.1
Non-Hispanic Asian, Native Hawaiian, or Pacific Islander	1.8	1.7	1.6	1.9	1.9	2.0
Non-Hispanic Multiracial	3.6	4.3	3.9	3.5	4.1	2.2
Non-Hispanic Other race^c^	0.5	0.7	0.3	0.5	0.5	0.6
Highest level of parental educational attainment (years), %						
≤ 12	15.3	20.9	18.6	16.1	12.4	9.8
13–15	25.2	27.4	27.6	25.9	24.1	21.9
16	29.0	26.3	27.8	29.9	30.5	30.9
≥ 17	30.5	25.4	26.1	28.1	33.0	37.4
Educational attainment (years), %						
≤ 12	4.9	8.3	7.1	4.2	3.1	2.5
13–15	19.8	29.1	23.3	21.3	16.3	11.3
16	32.4	27.6	31.7	34.7	34.0	33.9
≥ 17	42.9	35.1	37.9	39.7	46.7	52.3
Household income (U.S. dollars/year), %						
< $50,000	18.3	31.8	24.4	18.0	12.0	7.5
$50,000-$99,999	35.3	39.8	41.7	38.1	35.3	25.7
$100,000-$149,999	25.5	18.0	22.9	25.9	28.2	31.7
≥ $150,000	20.9	10.4	11.0	18.1	24.6	35.1
Current unemployment, %	14.0	17.2	15.8	13.2	13.3	10.8
Current body mass index (kg/m^2^), mean	28.0	29.7	29.0	28.2	27.3	26.3
Current smoker, %	8.5	12.5	11.5	9.0	5.9	4.4
≥ 7 Alcoholic drinks/week, %	13.5	12.1	12.2	13.8	14.8	14.1
Sleep duration < 7 h/night, %	23.9	28.2	25.8	25.3	22.8	18.7
Age at menarche < 12 years, %	24.9	28.2	25.8	24.0	24.4	22.7
Irregular cycles, %	16.0	19.6	17.6	15.3	13.7	13.8
Infrequent menstrual cycles (> 38 days), %	3.9	4.3	3.2	4.3	4.2	3.9
Frequent menstrual cycles (< 24 days), %	1.7	2.0	2.0	1.5	1.7	1.4
Gravid, %	51.8	55.3	53.3	51.9	50.1	48.9
Parous, %	34.3	35.5	36.0	35.8	32.3	31.9
Multivitamin use, %	80.2	72.7	79.0	79.9	83.4	85.2
Last method of contraception, %						
Oral contraceptives	31.6	31.5	30.7	32.1	32.0	32.1
Other hormonal methods	5.6	7.1	7.1	5.6	4.6	3.4
Barrier methods	41.8	38.0	39.7	42.6	43.7	44.2
Natural methods	21.0	23.4	22.6	19.7	19.8	20.3
Intercourse frequency < 1 time/week, %	22.1	20.1	21.9	21.9	22.2	23.2
Doing something to improve chances of conception, %	79.0	75.7	77.1	79.4	80.4	82.0
History of STI, %	13.3	17.9	14.4	13.1	12.7	9.5
History of infertility, %	8.9	13.6	10.4	8.2	7.4	5.4
History of uterine leiomyomata, %	2.3	2.9	1.8	2.4	2.3	2.2
History of endometriosis, %	3.1	3.1	3.0	3.2	3.7	2.3
History of polycystic ovary syndrome, %	9.0	10.4	9.3	9.8	7.8	7.7
≥ 1 visit to a primary care provider in the past year, %	86.7	84.7	86.0	87.8	87.5	87.0
High perceived stress (PSS score: ≥ 25), %	9.2	10.9	10.6	9.3	8.2	7.2
Severe depressive symptoms (MDI score: ≥ 30), %	4.7	7.0	5.4	5.0	3.6	2.9
< 3 cycles of attempt time at enrollment, %	66.6	60.9	63.2	67.5	68.3	71.6

*MDI* Major Depression Inventory, *PRESTO* Pregnancy Study Online, *PSS* Perceived Stress Scale, *Q* quintile, *STI* sexually transmitted infection (defined as chlamydia, genital herpes, or genital warts); ^a^Derived from the Index of Concentration at the Extremes; ^b^Standardized to the age distribution of the cohort at baseline; ^c^Includes American Indian, Alaskan Native, Indigenous, Middle Eastern or North African, some other race, or missing race

**Fig. 2:**
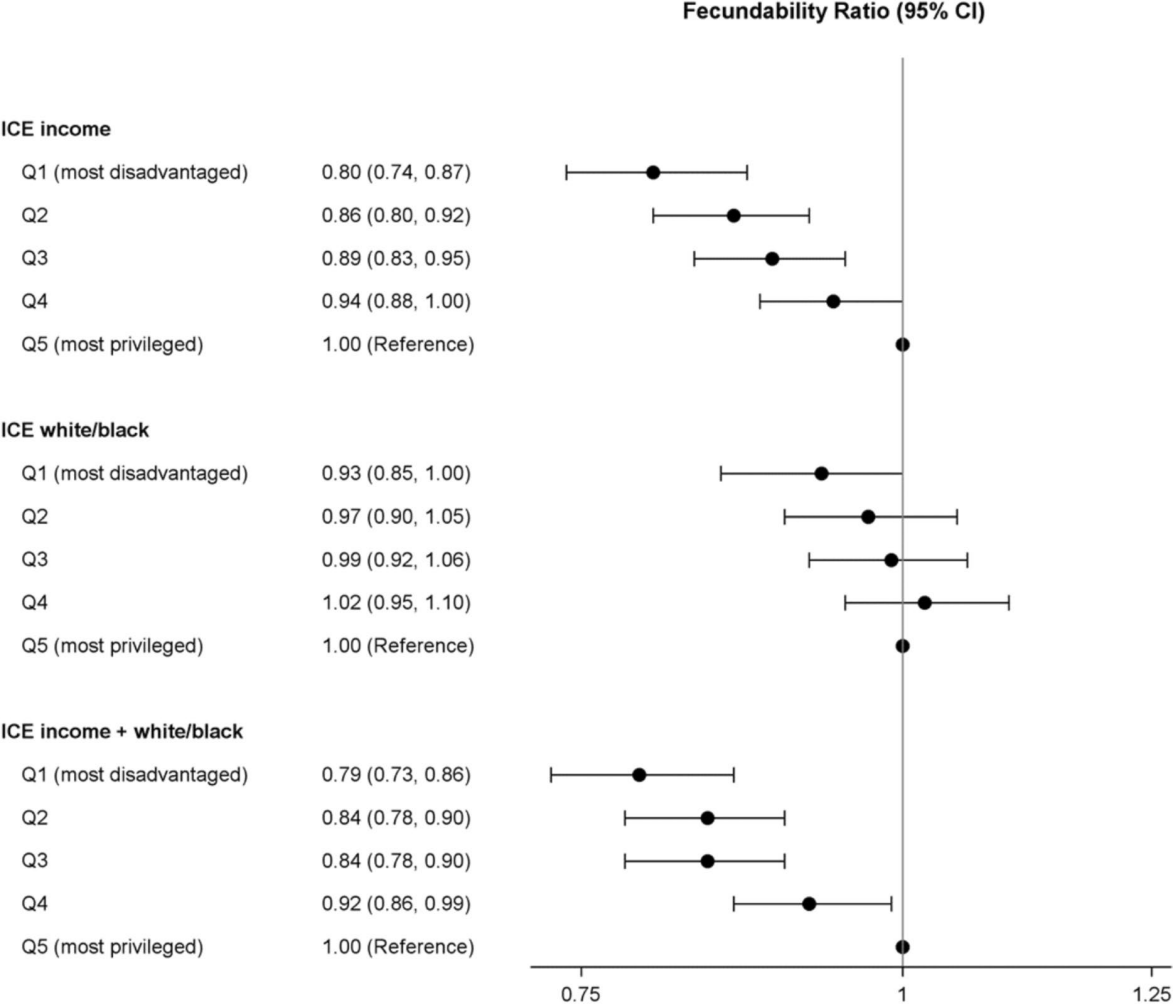
Associations between economic, racial, and racialized economic segregation with fecundability, PRESTO 2013–2022. Note: CI = confidence interval; ICE = Index of Concentration at the Extremes; PRESTO = Pregnancy Study Online; Adjusted for age, calendar year of enrollment, and geographic region of residence

**Fig. 3:**
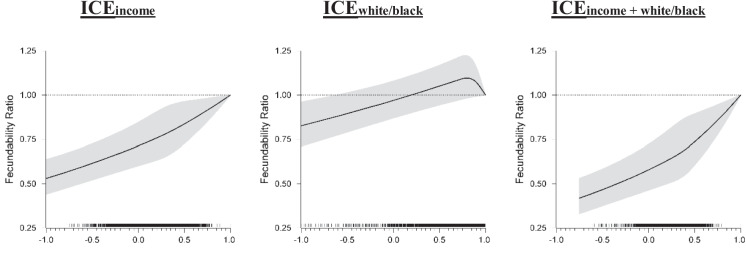
Restricted cubic splines for associations between economic, racial, and racialized economic segregation with fecundability, PRESTO 2013–2022. Note: PRESTO = Pregnancy Study Online; Graphs are plots of restricted cubic splines. Knots are located at the 50th, 75th, and 95th percentiles (ICE_income_: 0.1, 0.3, 0.6; ICE_white/black_: 0.7, 0.9, 1.0; ICE_income + white/black_: 0.2, 0.4, 0.5). The reference level is the maximum value of the exposure (score = 1.0). The black solid line indicates the fecundability ratio (FR) and the shaded gray area is the 95% confidence interval (CI); FRs are adjusted for age, calendar year of enrollment, and geographic region of residence. If the spline appears truncated, this signifies that the minimum value of exposure is greater than −1.0

We observed a monotonic inverse association between increasing quintiles of ICE_income_ and ICE_income + white/black_ with fecundability, but little evidence of an association between ICE_white/black_ and fecundability (Fig. 2;eTable 7). Compared with participants living in the most privileged neighborhoods with respect to racialized economic segregation, there is a 21% reduction in fecundability for participants living in the most disadvantaged neighborhoods (*i.e.,* FR for the most disadvantaged vs. most privileged quintile of ICE_income + white/black_). Our results were robust to alternative covariate adjustments, though attenuated (eTable 7). We observed little evidence of associations between ICE_white/hispanic_ and ICE_white/asian_ with fecundability (eFigure 3; eTable 7). Results based on restricted cubic splines were consistent with the categorical results, indicating declining fecundability as each ICE metric score decreased (Fig. 3;eFigure 4).

Associations across strata of pregnancy attempt time at enrollment were similar (eTable 8). We observed generally stronger results among parous participants (eTable 9) and those with a history of infertility (eTable 10). Associations were similar among nulligravid participants with < 3 cycles of pregnancy attempt time at enrollment (eTable 11). Some associations of segregation with fecundability were stronger among participants with high BMI (≥ 30 kg/m^2^; eTable 12**)**. Compared with non-Hispanic White participants, fecundability was appreciably lower for the most disadvantaged quintiles of all ICE metrics among participants that identified as non-Hispanic Other Race and Hispanic (eTable 13). We observed no appreciable difference by educational attainment (eTable 14) or household income (eTable 15). Results were similar when we restricted to participants residing in the same zip-code within the 12 months before enrollment and appended participants with a zip-code-level geocode to the analytic sample (eTable 16).

Mediation analyses showed that BMI ≥ 30 kg/m^2^ mediated the largest percentage of the association between racialized economic segregation (ICE_income + white/black_: quintile 1 vs. 5) and fecundability (25.8%), followed by < 16 years educational attainment (corresponding to a college degree: 21%; eTable 17). All other factors mediated ≤ 6.7% of the observed association.

## Discussion

Using data from a geographically diverse, internet-based cohort of pregnancy planners residing across the conterminous U.S, we observed a moderate association between living in the most disadvantaged neighborhoods with respect to racialized economic segregation and reduced fecundability. This association persisted after adjustment for individual-level confounders (*e.g.,* educational attainment, household income). We found some evidence of mediation by high BMI (25.8%) and lower educational attainment (21%). Associations between economic segregation (without accounting for racial influence) and fecundability were similar. These results support the scientific premise that economic segregation may be particularly influential for fertility, suggesting that the lack of resources in neighborhoods characterized as economically disadvantaged are more likely to be health-harming [74]. Unlike racialized economic segregation and economic segregation, racial segregation by itself (without accounting for economic influence) was not appreciably related to fecundability. Our body of results demonstrate the importance of considering the intersection of structural determinants that may drive disparities in fertility.

To our knowledge, no studies have examined segregation and fecundability. Nevertheless, our results align with existing literature on segregation and adverse pregnancy outcomes [10–12, 14–20]. One study in California showed evidence that economic, racial, and racialized economic segregation were all associated with preterm birth [19]. In contrast, recent work in Atlanta showed little evidence of an association between racialized economic segregation and any adverse birth outcome (*e.g.,* gestational age at delivery, birth weight, preterm birth, small for gestational age) among Black pregnant participants [13]. The inconsistency of associations across studies may reflect chance variation, but likely relate to differences in 1) study design, 2) study population, and/or 3) control for covariates that represent potential mediators [14, 75]. In addition, no studies have expanded ICE metrics for racial segregation (*i.e.,* ICE_white/hispanic_, ICE_white/asian_) and racialized economic segregation (*i.e.,* ICE_income + white/hispanic_, ICE_income + white/asian_), precluding a direct comparison to our results. More broadly, our results are consistent with the one existing study examining the effect of neighborhood disadvantage (defined by the Area Deprivation Index) on fecundability, which was conducted in the PRESTO cohort [30]. Since segregation is a root cause of racial and socioeconomic disparities, this study builds upon the existing literature to consider how segregation operates concurrently with neighborhood disadvantage.

Our findings of stronger associations among parous participants agree with previous work in PRESTO, the largest preconception cohort study conducted to date [30, 34, 35]. Parous participants with adequate financial resources may have more opportunity to change residence as their family grows [71–73]. Greater parity coupled with more material resources presents an opportunity for upward economic mobility and relocation to areas with more favorable neighborhood conditions (*e.g.,* close proximity to health care), which are referred to as “high-opportunity neighborhoods.” High-opportunity neighborhoods have positive effects on residents’ well-being (*e.g.,* allostatic load [76], inflammation [77]) and potential benefits from racialized economic patterns of neighborhood investment (*e.g.,* improved access to health care). Even so, we acknowledge these relations are not deterministic. An individual’s life chances are driven and influenced by the neighborhood environments in which they have grown up and continue to live in or relocate to [78–80]. For example, residence in a disadvantaged neighborhood may reduce socioeconomic mobility. Moreover, individuals with less education and income may have less access to well-resourced neighborhoods, influencing their choice (or lack thereof) to live in a certain neighborhood based on its racial or economic composition.

Mixed findings across studies may be explained by differences in absolute and relative income, such as income incongruity (defined as the extent to which an individual resides in a poorer or wealthier neighborhood than expected based on others with the same educational attainment and marital status) [81, 82]. Empirical evidence indicates income incongruity may reflect a distinct dimension of disadvantage or privilege within neighborhoods. For instance, a previous study observed the beneficial effect of positive income incongruity on pregnancy outcomes was only evident among Black women living in census tracts with high racial density (*i.e.,* over 90% Black residents) [83]. Since the majority of PRESTO participants identify as non-Hispanic White, our results may not capture some of these subtle differences in the influence of racialized economic segregation for specific subgroups (*e.g.,* Black participants).

Segregation is a complex phenomenon comprised of five dimensions [2, 84]. While we are not aware of any segregation measure that addresses all five dimensions, the ICE is designed to simultaneously account for polarization in disadvantaged and privileged neighborhoods while jointly measuring economic and racial segregation [51], overcoming limitations of other measures (*e.g.,* Index of Dissimilarity [racial segregation], Gini Coefficient [income inequality]) that 1) only assess evenness [2, 75], 2) cannot show directionality of the concentration within the distribution (*i.e.,* −1.0 to 1.0) [48], and 3) are argued to be less informative at lower levels of geography (*e.g.,* census tract, block group) [51, 75]. The ICE can be meaningfully computed at varying spatial scales [48, 49], with smaller geographic units (*e.g.,* census tracts) as most optimal. A key challenge of investigating racialized economic segregation, however, lies in its formulation, which is shaped by factors operating at varied levels (Fig. 1) [1]. Many of these factors are also influenced by historical policies and practices, such as redlining, that resulted in neighborhood (dis)investment, contributing to concentrated disadvantage and place-based disparities [85].

Segregation also creates differential exposure to social and economic resources, constraining or expanding individual-level opportunity structures that affect current health and health trajectories [86, 87]. The effects of segregation are best acknowledged as dual-faceted, reflecting both negative (*e.g.,* poverty, pollution, urban blight) and positive consequences (*e.g.,* cultural preservation, reduced discrimination, shared resources) [88]. In our study, all restricted cubic splines for racial segregation showed shorter TTP in the privileged extremes.

Our mediation analysis facilitated the investigation of several pathways that could inform targeted interventions to improve population-level fertility. Most notably, we observed high BMI (≥ 30 kg/m^2^) and lower educational attainment (< 16 years) explained meaningful percentages of the association between segregation and fecundability. Extreme disadvantage of racialized economic segregation may contribute to obesogenic environments (*e.g.,* limited selection of fresh fruits and vegetables, reduced access to supermarkets, higher density of fast food restaurants) [89–91]. Health-harming segregated neighborhoods may also be perceived as less safe (*e.g.,* higher crime) and include limited infrastructure (*e.g.,* recreational facilities, parks, sidewalks) that promote physical activity accompanying a healthy diet [2]. Regarding the largely mediated effect of lower educational attainment, segregation determines access to educational opportunities and schools are sometimes viewed as the “hub and heart” of a neighborhood environment [1, 2], aligning with our results. Further, one study showed segregation has a measurable influence on the educational attainment for Black but not White participants [92]. In contrast, mediation results indicated current unemployment status and participants’ mental health (*e.g.,* perceived stress) may not exert strong indirect effects between segregation and fecundability. Although selected indicators of physiological reactivity (*e.g.,* heightened activity in the hypothalamic–pituitary–adrenal axis, elevated cortisol levels, allostatic load or “weathering” [75, 93]) are valuable areas of research pursuit and intervention, the observed associations suggest these pathways may be less likely to serve as mediators in this context. Collectively, the results from the exploratory mediation analyses support the hypothesis that racialized economic segregation can influence the physical and social environment, embodied neighborhood experiences, and individual opportunity or exposure.

### Limitations

We assessed segregation based on geocoded residential addresses reported at baseline, which did not allow us to account for participants’ housing characteristics or time-activity patterns [94, 95]. However, the prospective study design allows us to ascertain residential addresses before pregnancy [30, 35]; therefore, exposure misclassification with respect to the outcome is likely nondifferential. This cohort also has low residential mobility (2.3% of participants reported relocating over follow up), and our results were comparable to the main analysis when we restricted to participants who have the same zip-code within the 12 months prior to enrollment. Moreover, potential concern of “spillover effects” via participants accessing resources in adjacent neighborhoods is minimal as prior research indicates ICE metrics should be implemented at the census tract level (as opposed to city/town) to avoid underestimation [49].

Fecundability was calculated based on self-reported menstrual data captured prospectively over time. Other work in this cohort indicates high reliability of menstruation data [44], and that > 96% of participants use home pregnancy tests for early detection [96]. Although outcome misclassification is possible, it is unlikely related to segregation.

Our mediation analysis did not account for potential interactions among mediators as we examined them separately (*i.e.,* single-mediator analyses) [97]. Current approaches for mediator-mediator interactions require confirmation of all mediators along the pathway to assess them sequentially [98]. Some mediators (*e.g.,* air pollution) may even operate more strongly as effect modifiers [99]. Although all mediators explored were ascertained at baseline, we decided this was reasonable because 1) we conceptualize these individual-level factors to be downstream effects of the neighborhood environment (“occurring after segregation”; Fig. 1), 2) some characteristics are only ascertained at baseline (*e.g.,* BMI), and 3) many characteristics do not vary appreciably with increasing pregnancy attempt time in our cohort (*i.e.,* perceived stress scores at 12 months after baseline) [58].

Results derived from a cohort of pregnancy planners may not be generalizable to the general U.S. population [100, 101]. Many PRESTO participants report higher socioeconomic status [24], and 83% identifies as non-Hispanic White. We also recognize there are stark differences in the lived experience among Black, Indigenous, and People of Color, thus there are limitations in our interpretation using broad groupings. Because segregation is a manifestation of structural racism, we hypothesize that stratification by more granular categories of race/ethnicity in regression models may have revealed additional insights, building upon the strata explored in our study.

We acknowledge the possibility of “neighborhood self-selection” (defined as individuals choosing to live in certain neighborhoods because they offer resources that optimize health) [74, 80, 102]. To explore this, we examined potential modification of the segregation-fecundability association by parity, educational attainment, and household income (*i.e.,* determinants of neighborhood self-selection) to evaluate, in part, the extent to which neighborhood self-selection influenced our results. In the present study, we observed stronger associations among parous participants, but relatively uniform associations across categories of educational attainment and household income.

## Conclusion

Living in the most disadvantaged neighborhoods with respect to racialized economic segregation was associated with a moderate decrease in fecundability. Our findings underscore the importance of investigating structural determinants of fertility, including potential mechanisms that may explain this association. While neighborhood disadvantage is recognized as a key determinant of racial and socioeconomic disparities in reproductive health, there is limited exploration of segregation (the root cause of such disparities) in the fertility literature. Regardless of potential causality, programs or policies that improve neighborhood environments could reduce population-level burden of infertility and advance health equity, particularly among participants who reside in neighborhoods that have been historically and currently marginalized.

## Supplementary Information

Below is the link to the electronic supplementary material.

Supplementary file1 (DOCX 785 KB)

## Data Availability

PRESTO participants did not give informed consent to share data with external researchers.

## Abbreviations

*BMI*: Body mass index
*CI*: Confidence interval
*IQR*: Interquartile range
*FR*: Fecundability ratio
*LMP*: Last menstrual period
*MDI*: Major Depression Inventory
*PRESTO*: Pregnancy Study Online
*PSS*: Perceived Stress Scale
*Q*: Quintile
*SD*: Standard deviation
*STI*: Sexually transmitted infection
*TTP*: Time-to-pregnancy
