# Supplementary material for: Racialized economic segregation in relation to fecundability in a preconception cohort study
Source: J Urban Health. 2025 Dec 20;103(1):104–19. doi: 10.1007/s11524-025-01038-y (PMC13056346; doi:10.1007/s11524-025-01038-y)
Supplement: Supplementary file 1 — Supplementary file1 (DOCX 785 KB) [file 11524_2025_1038_MOESM1_ESM.docx]

## **Supplementary Material**

**Racialized economic segregation in relation to fecundability in a preconception cohort study**

Sharonda M. Lovett, Erin J. Campbell, Andrea S. Richardson, Amelia K. Wesselink,

Collette N. Ncube, Yvette C. Cozier, Lauren A. Wise, & Mary D. Willis

**Table of Contents**

**eTable 1.** Calculation of the Index of Concentration at the Extremes (ICE) metrics using 5-year estimates from the American Community Survey (ACS), 2013-2022

**eTable 2.** Distribution of ICE metrics within quintiles in PRESTO

**eFigure 1.** Data flow chart

**eTable 3.** Characteristics of 10,438 participants by loss to follow-up, PRESTO 2013-2022

**eFigure 2.** Mapped distribution of PRESTO participants in the analytic sample across U.S. states

**eTable 4.** Demographic characteristics stratified by economic segregation and racial segregation, PRESTO 2013-2022

**eTable 5.** Distribution of ICE metrics in PRESTO, overall and stratified by race/ethnicity

**eTable 6.** Correlation matrix^a^ of ICE metrics, PRESTO 2013-2022

**eTable 7.** Associations between economic, racial, and racialized economic segregation with fecundability, PRESTO 2013-2022

**eFigure 3.** Associations between other metrics of economic, racial, and racialized economic segregation with fecundability, PRESTO 2013-2022

**eFigure 4.** Restricted cubic splines for associations between other metrics of economic, racial, and racialized economic segregation with fecundability, PRESTO 2013-2022

**eTable 8.** Associations between economic, racial, and racialized economic segregation with fecundability stratified by pregnancy attempt time at enrollment, PRESTO 2013-2022

**eTable 9.** Associations between economic, racial, and racialized economic segregation with fecundability stratified by parity, PRESTO 2013-2022

**eTable 10.** Associations between economic, racial, and racialized economic segregation with fecundability stratified by infertility history, PRESTO 2013-2022

**eTable 11.** Associations between economic, racial, and racialized economic segregation with fecundability among nulligravid participants with <3 cycles of pregnancy attempt time at enrollment, overall and further restricted to participants without a history of infertility, PRESTO 2013-2022

**eTable 12.** Associations between economic, racial, and racialized economic segregation with fecundability stratified by body mass index, PRESTO 2013-2022

**eTable 13.** Associations between economic, racial, and racialized economic segregation with fecundability stratified by participants’ race/ethnicity, PRESTO 2013-2022

**eTable 14.** Associations between economic, racial, and racialized economic segregation with fecundability stratified by educational attainment, PRESTO 2013-2022

**eTable 15.** Associations between economic, racial, and racialized economic segregation with fecundability stratified by household income, PRESTO 2013-2022

**eTable 16.** Associations between economic, racial, and racialized economic segregation with fecundability accounting for adjustments in the analytic sample related to residential mobility and geospatial exposure assessment, PRESTO 2013-2022

**eTable 17.** Natural direct and indirect effects of racialized economic segregation (ICE_income + white/black_) in the most disadvantaged (Q1) vs. most privileged (Q5) quintile and fecundability, PRESTO 2013-2022

**eTable 1.** Calculation of the Index of Concentration at the Extremes (ICE) metrics using 5-year estimates from the American Community Survey (ACS), 2013-2022

| **Domain** | **Formula** | **ACS Table ID** |
| --- | --- | --- |
| ICE_education_ | ((≥16 years)-(<12 years))/Total | B15003^a^ |
| ICE_income_ | ((≥$100k)-(<$25k))/Total | B19001 |
| ICE_white/black_ | ((non-Hispanic White)-(non-Hispanic Black))/Total | B03002 |
| ICE_white/hispanic_ | ((non-Hispanic White)-(Hispanic))/Total | B03002 |
| ICE_white/asian_ | ((non-Hispanic White)-(non-Hispanic Asian^b^))/Total | B03002 |
| ICE_income + white/black_ | ((non-Hispanic White ≥$100k)-(non-Hispanic Black <$25k))/Total | B19001A-B19001H |
| ICE_income + white/hispanic_ | ((non-Hispanic White ≥$100k)-(Hispanic <$25k))/Total | B19001A-B19001H |
| ICE_income + white/asian_ | ((non-Hispanic White ≥$100k)-(non-Hispanic Asian^b^ <$25k))/Total | B19001A-B19001H |

Note: Shapefiles were downloaded from the National Historical Geographic Information System (<https://www.nhgis.org/>); k = thousand; ^a^Determined solely among adults aged ≥25 years; ^b^Defined as “Asian Alone”

**eTable 2.** Distribution of ICE metrics within quintiles in PRESTO

|  | **Mean (SD)** | **Median (IQR)** | **Minimum** | **Maximum** |
| --- | --- | --- | --- | --- |
| **ICE_income_** |  |  |  |  |
| Q1 (most disadvantaged) | -0.3 (0.1) | -0.2 (-0.3, -0.2) | -1.0 | -0.1 |
| Q2 | 0.0 (0.0) | 0.0 (-0.1, 0.0) | -0.1 | 0.0 |
| Q3 | 0.1 (0.0) | 0.1 (0.1, 0.2) | 0.0 | 0.2 |
| Q4 | 0.3 (0.0) | 0.3 (0.2, 0.3) | 0.2 | 0.4 |
| Q5 (most privileged) | 0.5 (0.1) | 0.5 (0.4, 0.6) | 0.4 | 1.0 |
| **ICE_white/black_** |  |  |  |  |
| Q1 (most disadvantaged) | 0.0 (0.3) | 0.1 (-0.1, 0.2) | -1.0 | 0.3 |
| Q2 | 0.5 (0.1) | 0.5 (0.4, 0.6) | 0.3 | 0.6 |
| Q3 | 0.7 (0.0) | 0.7 (0.7, 0.7) | 0.6 | 0.8 |
| Q4 | 0.8 (0.0) | 0.8 (0.8, 0.9) | 0.8 | 0.9 |
| Q5 (most privileged) | 0.9 (0.0) | 0.9 (0.9, 1.0) | 0.9 | 1.0 |
| **ICE_income + white/black_** |  |  |  |  |
| Q1 (most disadvantaged) | 0.0 (0.1) | 0.0 (-0.1, 0.0) | -0.7 | 0.1 |
| Q2 | 0.1 (0.0) | 0.1 (0.1, 0.1) | 0.1 | 0.2 |
| Q3 | 0.2 (0.0) | 0.2 (0.2, 0.2) | 0.2 | 0.3 |
| Q4 | 0.3 (0.0) | 0.3 (0.3, 0.3) | 0.3 | 0.4 |
| Q5 (most privileged) | 0.5 (0.1) | 0.5 (0.4, 0.5) | 0.4 | 1.0 |
| **ICE_education_** |  |  |  |  |
| Q1 (most disadvantaged) | -0.1 (0.1) | 0.0 (-0.1, 0.0) | -0.6 | 0.0 |
| Q2 | 0.1 (0.0) | 0.1 (0.1, 0.1) | 0.0 | 0.2 |
| Q3 | 0.3 (0.0) | 0.2 (0.2, 0.3) | 0.2 | 0.3 |
| Q4 | 0.4 (0.1) | 0.4 (0.4, 0.5) | 0.3 | 0.5 |
| Q5 (most privileged) | 0.6 (0.1) | 0.6 (0.6, 0.7) | 0.5 | 1.0 |
| **ICE_white/hispanic_** |  |  |  |  |
| Q1 (most disadvantaged) | 0.0 (0.3) | 0.1 (-0.1, 0.2) | -1.0 | 0.3 |
| Q2 | 0.5 (0.1) | 0.5 (0.4, 0.5) | 0.3 | 0.6 |
| Q3 | 0.7 (0.0) | 0.7 (0.6, 0.7) | 0.6 | 0.7 |
| Q4 | 0.8 (0.0) | 0.8 (0.8, 0.8) | 0.7 | 0.9 |
| Q5 (most privileged) | 0.9 (0.0) | 0.9 (0.9, 0.9) | 0.9 | 1.0 |
| **ICE_white/asian_** |  |  |  |  |
| Q1 (most disadvantaged) | 0.2 (0.2) | 0.2 (0.1, 0.3) | -0.7 | 0.4 |
| Q2 | 0.5 (0.1) | 0.5 (0.5, 0.6) | 0.4 | 0.6 |
| Q3 | 0.7 (0.0) | 0.7 (0.7, 0.7) | 0.6 | 0.8 |
| Q4 | 0.8 (0.0) | 0.8 (0.8, 0.9) | 0.8 | 0.9 |
| Q5 (most privileged) | 0.9 (0.0) | 0.9 (0.9, 1.0) | 0.9 | 1.0 |
| **ICE_income + white/hispanic_** |  |  |  |  |
| Q1 (most disadvantaged) | -0.3 (0.1) | -0.3 (-0.4, -0.2) | -1.0 | -0.1 |
| Q2 | -0.1 (0.0) | -0.1 (-0.1, 0.0) | -0.1 | 0.0 |
| Q3 | 0.1 (0.0) | 0.1 (0.0, 0.1) | 0.0 | 0.1 |
| Q4 | 0.2 (0.0) | 0.2 (0.2, 0.2) | 0.1 | 0.3 |
| Q5 (most privileged) | 0.4 (0.1) | 0.4 (0.3, 0.5) | 0.3 | 1.0 |
| **ICE_income + white/asian_** |  |  |  |  |
| Q1 (most disadvantaged) | 0.0 (0.0) | 0.0 (0.0, 0.1) | -0.3 | 0.1 |
| Q2 | 0.1 (0.0) | 0.1 (0.1, 0.2) | 0.1 | 0.2 |
| Q3 | 0.2 (0.0) | 0.2 (0.2, 0.2) | 0.2 | 0.3 |
| Q4 | 0.3 (0.0) | 0.3 (0.3, 0.3) | 0.3 | 0.4 |
| Q5 (most privileged) | 0.5 (0.1) | 0.5 (0.4, 0.5) | 0.4 | 1.0 |

Note: ICE = Index of Concentration at the Extremes; IQR = interquartile range; PRESTO = Pregnancy Study Online; SD = standard deviation

**eFigure 1.** Data flow chart


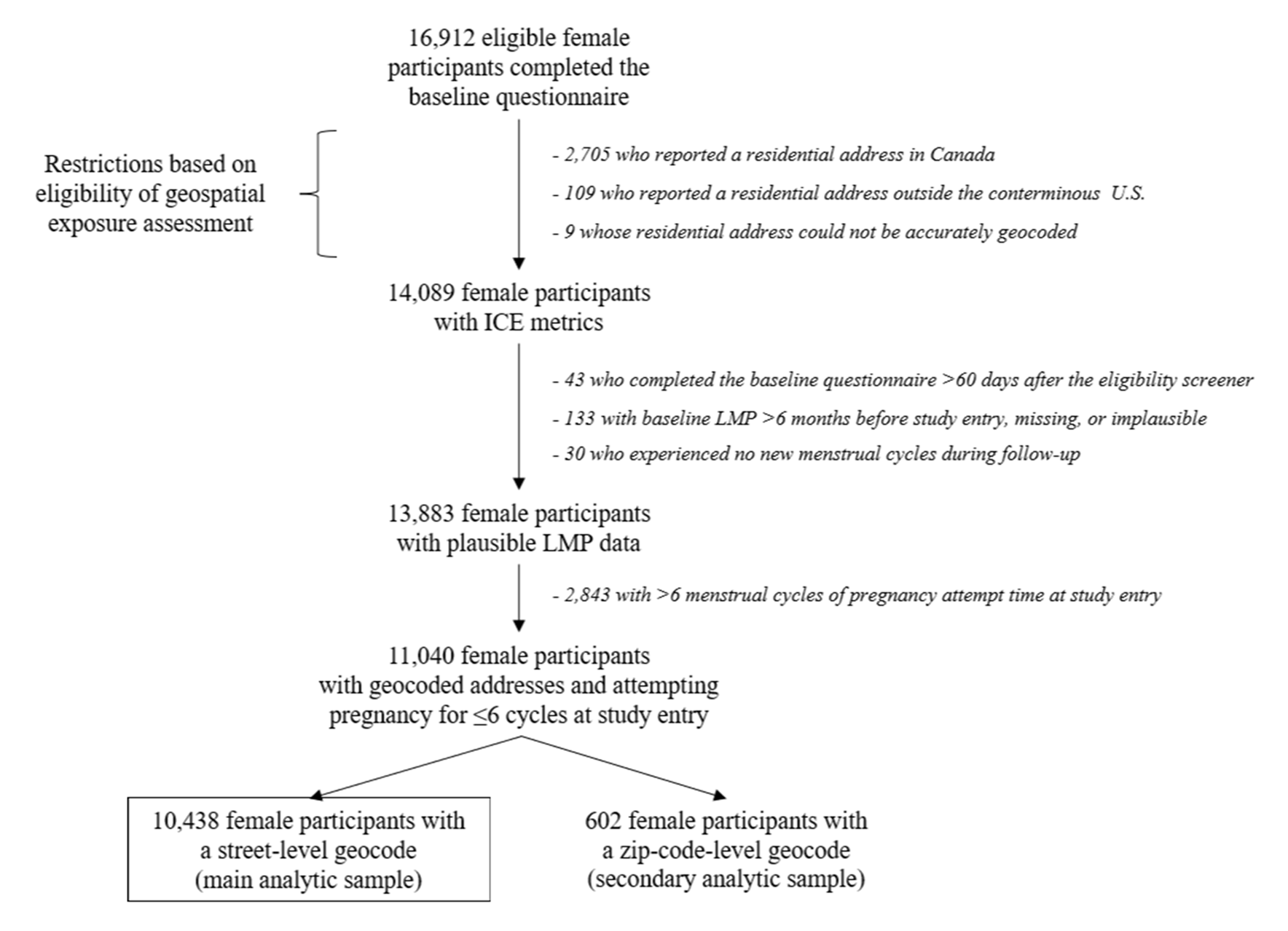


Note: ICE = Index of Concentration at the Extremes; LMP = last menstrual period

**eTable 3.** Characteristics of 10,438 participants by loss to follow-up, PRESTO 2013-2022

| Characteristic^a^ | **Lost to Follow-up** | **Not Lost to Follow-up** |
| --- | --- | --- |
|  | (n=1,893) | (n=8,545) |
| Age (years), mean | 30.0 | 30.3 |
| Married, % | 79.2 | 92.1 |
| Residence in an urban area, % | 92.6 | 95.5 |
| Geographic region of residence, % |  |  |
| U.S. Northeast | 17.7 | 28.2 |
| U.S. South | 33.4 | 26.6 |
| U.S. Midwest | 28.0 | 26.2 |
| U.S. West | 21.0 | 19.0 |
| Race/ethnicity, % |  |  |
| Non-Hispanic White | 75.7 | 85.1 |
| Non-Hispanic Black | 7.6 | 2.4 |
| Hispanic | 9.8 | 6.9 |
| Non-Hispanic Asian, Native Hawaiian, or Pacific Islander | 2.3 | 1.7 |
| Non-Hispanic Multiracial | 4.2 | 3.5 |
| Non-Hispanic Other race^c^ | 0.6 | 0.5 |
| Highest level of parental educational attainment (years), % |  |  |
| ≤12 | 24.4 | 13.2 |
| 13-15 | 29.4 | 24.4 |
| 16 | 25.8 | 29.8 |
| ≥17 | 20.3 | 32.6 |
| Educational attainment (years), % |  |  |
| ≤12 | 12.1 | 3.3 |
| 13-15 | 33.4 | 16.8 |
| 16 | 28.2 | 33.5 |
| ≥17 | 26.4 | 46.4 |
| Household income (U.S. dollars/year), % |  |  |
| <$50,000 | 31.7 | 15.5 |
| $50,000-$99,999 | 35.1 | 35.3 |
| $100,000-$149,999 | 21.1 | 26.5 |
| ≥$150,000 | 12.0 | 22.7 |
| Current unemployment, % | 18.4 | 13.1 |
| Current body mass index (kg/m^2^), mean | 31.3 | 27.3 |
| Current smoker, % | 15.5 | 7.0 |
| ≥7 Alcoholic drinks/week, % | 11.0 | 13.9 |
| Sleep duration <7 hours/night, % | 33.1 | 21.9 |
| Age at menarche <12 years, % | 29.5 | 23.9 |
| Irregular cycles, % | 26.3 | 13.7 |
| Infrequent menstrual cycles (>38 days), % | 7.1 | 3.2 |
| Frequent menstrual cycles (<24 days), % | 2.6 | 1.5 |
| Gravid, % | 58.3 | 50.3 |
| Parous, % | 40.0 | 33.0 |
| Multivitamin use, % | 70.1 | 82.3 |
| Last method of contraception, % |  |  |
| Oral contraceptives | 29.6 | 32.1 |
| Other hormonal methods | 8.6 | 4.8 |
| Barrier methods | 36.1 | 43.0 |
| Natural methods | 25.7 | 20.1 |
| Intercourse frequency <1 time/week, % | 24.8 | 21.5 |
| Doing something to improve chances of conception, % | 75.4 | 79.7 |
| History of STI, % | 16.4 | 12.6 |
| History of infertility, % | 19.1 | 6.6 |
| History of uterine leiomyomata, % | 2.7 | 2.2 |
| History of endometriosis, % | 3.7 | 3.0 |
| History of polycystic ovary syndrome, % | 16.4 | 7.4 |
| ≥1 visit to a primary care provider in the past year, % | 86.9 | 86.6 |
| High perceived stress (PSS score: ≥25), % | 13.3 | 8.2 |
| Severe depressive symptoms (MDI score: ≥30), % | 9.6 | 3.6 |
| <3 cycles of attempt time at enrollment, % | 56.6 | 68.9 |

Note: MDI = Major Depression Inventory; PRESTO = Pregnancy Study Online; PSS = Perceived Stress Scale; STI = sexually transmitted infection (defined as chlamydia, genital herpes, or genital warts); ^a^Standardized to the age distribution of the cohort at baseline; ^c^Includes American Indian, Alaskan Native, Indigenous, Middle Eastern or North African, some other race, or missing race.

**eFigure 2.** Mapped distribution of PRESTO participants in the analytic sample across U.S. states

**
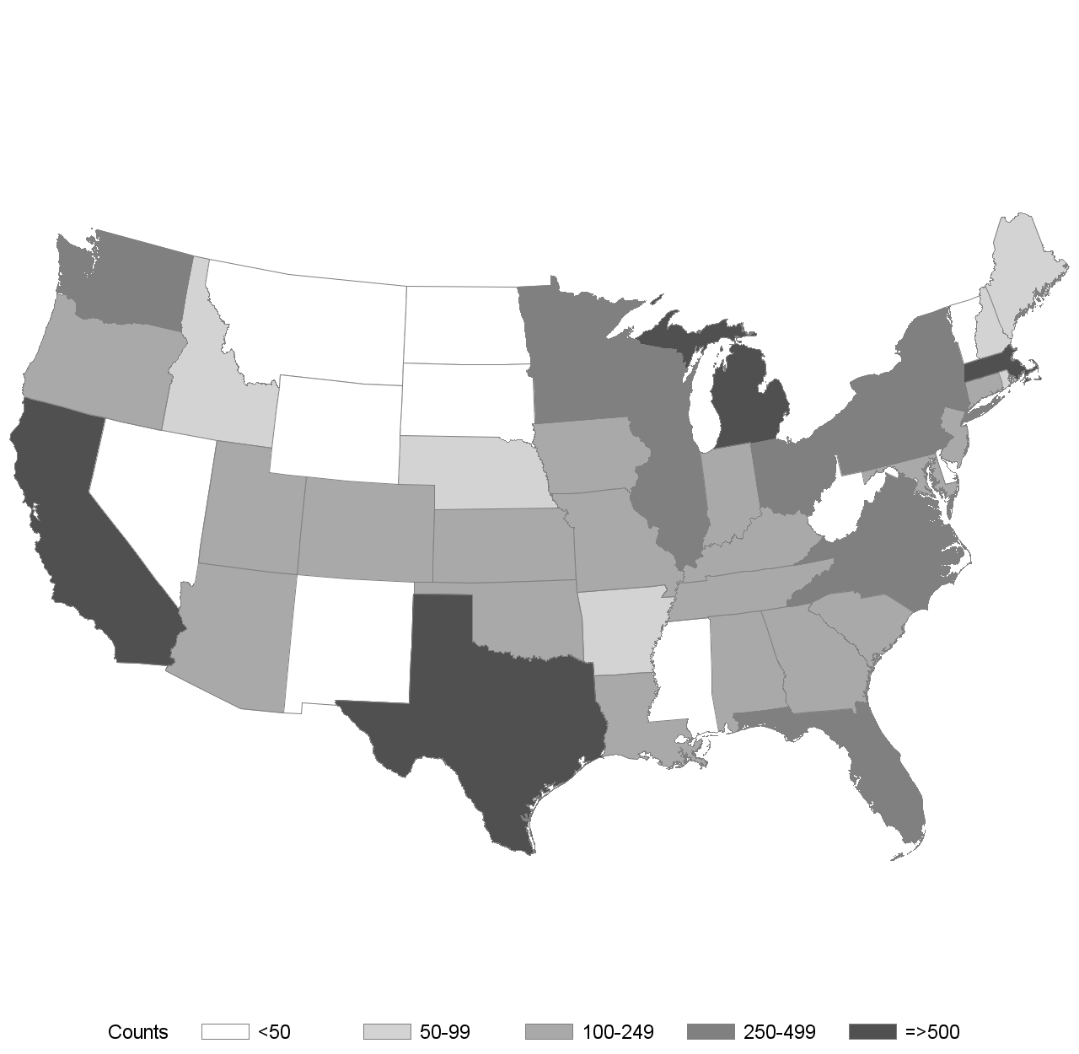
**

Note: PRESTO = Pregnancy Study Online

**eTable 4.** Demographic characteristics stratified by economic segregation and racial segregation, PRESTO 2013-2022

| Characteristic^b^ | **Economic segregation (ICE_income_ score)**^a^ | | | | | **Racial segregation (ICE_white/black_ score)**^a^ | | | | |
| --- | --- | --- | --- | --- | --- | --- | --- | --- | --- | --- |
|  | Q1  (most disadvantaged) | Q2 | Q3 | Q4 | Q5  (most privileged) | Q1  (most disadvantaged) | Q2 | Q3 | Q4 | Q5  (most privileged) |
|  | (n=1,825) | (n=1,996) | (n=2,148) | (n=2,195) | (n=2,274) | (n=1,918) | (n=2,135) | (n=2,183) | (n=2,164) | (n=2,038) |
| Age (years), mean | 29.4 | 29.7 | 30.1 | 30.7 | 31.1 | 30.4 | 30.4 | 30.3 | 30.3 | 29.7 |
| Married, % | 83.7 | 88.0 | 89.5 | 91.9 | 93.8 | 84.3 | 91.8 | 91.4 | 91.5 | 89.4 |
| Residence in an urban area, % | 93.8 | 91.2 | 93.9 | 96.8 | 98.7 | 98.7 | 97.9 | 97.7 | 96.6 | 84.1 |
| Geographic region of residence, % |  |  |  |  |  |  |  |  |  |  |
| U.S. Northeast | 19.1 | 20.5 | 27.0 | 29.1 | 33.4 | 16.4 | 21.2 | 26.5 | 27.7 | 39.5 |
| U.S. South | 37.0 | 30.4 | 25.3 | 23.6 | 25.1 | 48.2 | 35.4 | 25.7 | 19.2 | 12.2 |
| U.S. Midwest | 28.2 | 30.1 | 28.5 | 27.0 | 19.5 | 15.3 | 18.7 | 22.6 | 33.1 | 42.1 |
| U.S. West | 15.7 | 19.0 | 19.3 | 20.3 | 22.0 | 20.1 | 24.7 | 25.3 | 20.0 | 6.2 |
| Race/ethnicity, % |  |  |  |  |  |  |  |  |  |  |
| Non-Hispanic White | 78.3 | 83.8 | 83.7 | 85.6 | 84.4 | 68.4 | 82.3 | 83.9 | 89.5 | 92.2 |
| Non-Hispanic Black | 7.7 | 3.8 | 2.7 | 1.5 | 1.9 | 10.9 | 2.5 | 1.9 | 0.9 | 0.8 |
| Hispanic | 8.5 | 6.8 | 7.3 | 7.1 | 7.5 | 13.0 | 8.6 | 7.7 | 4.6 | 3.5 |
| Non-Hispanic Asian, Native Hawaiian, or Pacific Islander | 1.1 | 1.4 | 1.6 | 2.1 | 2.6 | 2.8 | 2.4 | 2.0 | 1.3 | 0.7 |
| Non-Hispanic Multiracial | 3.7 | 3.9 | 4.3 | 3.4 | 2.9 | 4.4 | 3.6 | 4.0 | 3.4 | 2.2 |
| Non-Hispanic Other race^c^ | 0.7 | 0.4 | 0.5 | 0.3 | 0.7 | 0.6 | 0.6 | 0.6 | 0.4 | 0.5 |
| Highest level of parental educational attainment (years), % |  |  |  |  |  |  |  |  |  |  |
| ≤12 | 23.2 | 17.5 | 15.2 | 12.5 | 9.9 | 17.6 | 13.2 | 12.7 | 14.8 | 18.3 |
| 13-15 | 29.7 | 27.3 | 25.7 | 24.8 | 20.0 | 25.0 | 24.0 | 24.2 | 24.4 | 28.6 |
| 16 | 25.1 | 28.7 | 29.4 | 30.1 | 31.7 | 27.1 | 28.9 | 31.7 | 28.5 | 29.0 |
| ≥17 | 22.0 | 26.6 | 29.7 | 32.6 | 38.5 | 30.2 | 33.9 | 31.5 | 32.3 | 24.2 |
| Educational attainment (years), % |  |  |  |  |  |  |  |  |  |  |
| ≤12 | 9.7 | 6.0 | 4.7 | 2.7 | 2.4 | 6.6 | 4.1 | 4.0 | 4.1 | 6.0 |
| 13-15 | 30.3 | 24.6 | 19.3 | 15.7 | 12.0 | 23.8 | 17.0 | 18.3 | 18.0 | 22.4 |
| 16 | 27.6 | 32.1 | 34.1 | 34.9 | 33.3 | 28.5 | 31.9 | 33.9 | 33.5 | 34.0 |
| ≥17 | 32.5 | 37.4 | 42.0 | 46.7 | 52.3 | 41.2 | 47.1 | 43.8 | 44.5 | 37.7 |
| Household income (U.S. dollars/year), % |  |  |  |  |  |  |  |  |  |  |
| <$50,000 | 35.6 | 24.2 | 16.1 | 11.3 | 7.4 | 24.3 | 15.1 | 17.1 | 16.3 | 19.7 |
| $50,000-$99,999 | 41.5 | 41.5 | 40.3 | 34.6 | 23.4 | 35.2 | 35.7 | 32.0 | 34.9 | 38.9 |
| $100,000-$149,999 | 15.8 | 23.4 | 26.0 | 29.8 | 31.3 | 21.4 | 25.5 | 26.9 | 27.4 | 25.8 |
| ≥$150,000 | 7.2 | 10.9 | 17.5 | 24.3 | 37.9 | 19.1 | 23.7 | 24.0 | 21.4 | 15.6 |
| Current unemployment, % | 18.0 | 14.7 | 13.5 | 12.5 | 12.1 | 16.6 | 13.3 | 14.5 | 12.5 | 13.9 |
| Current body mass index (kg/m^2^), mean | 30.1 | 28.9 | 28.1 | 27.3 | 26.2 | 28.6 | 27.9 | 27.5 | 27.7 | 28.5 |
| Current smoker, % | 14.5 | 10.6 | 8.5 | 6.0 | 4.1 | 9.3 | 7.1 | 8.0 | 7.5 | 10.5 |
| ≥7 Alcoholic drinks/week, % | 11.1 | 13.8 | 13.4 | 14.2 | 14.2 | 13.7 | 12.1 | 13.6 | 14.4 | 13.6 |
| Sleep duration <7 hours/night, % | 29.5 | 27.0 | 23.2 | 22.0 | 19.8 | 26.3 | 23.6 | 22.1 | 21.7 | 26.2 |
| Age at menarche <12 years, % | 28.6 | 25.4 | 24.5 | 24.2 | 22.6 | 27.2 | 26.3 | 24.3 | 22.9 | 24.3 |
| Irregular cycles, % | 20.5 | 17.0 | 14.7 | 14.5 | 13.5 | 18.1 | 15.8 | 15.1 | 14.8 | 16.5 |
| Infrequent menstrual cycles (>38 days), % | 4.2 | 3.5 | 3.7 | 4.3 | 4.3 | 4.2 | 3.9 | 3.8 | 4.0 | 3.8 |
| Frequent menstrual cycles (<24 days), % | 2.3 | 1.6 | 1.9 | 1.6 | 1.2 | 2.0 | 1.4 | 1.7 | 1.5 | 2.1 |
| Gravid, % | 57.3 | 52.6 | 51.6 | 49.9 | 48.3 | 52.7 | 48.8 | 49.2 | 52.4 | 56.1 |
| Parous, % | 39.4 | 35.1 | 33.6 | 33.0 | 31.0 | 32.4 | 29.9 | 33.5 | 36.5 | 39.1 |
| Multivitamin use, % | 72.6 | 77.7 | 80.5 | 83.3 | 85.5 | 75.3 | 81.7 | 80.8 | 82.2 | 80.7 |
| Last method of contraception, % |  |  |  |  |  |  |  |  |  |  |
| Oral contraceptives | 30.5 | 31.7 | 33.5 | 31.5 | 31.0 | 29.8 | 32.1 | 31.8 | 32.2 | 31.6 |
| Other hormonal methods | 7.8 | 6.3 | 5.7 | 3.9 | 4.2 | 6.0 | 5.5 | 5.3 | 4.6 | 6.4 |
| Barrier methods | 37.6 | 40.7 | 40.4 | 45.4 | 43.9 | 42.3 | 41.9 | 42.5 | 42.6 | 40.1 |
| Natural methods | 24.1 | 21.3 | 20.4 | 19.3 | 20.9 | 22.0 | 20.5 | 20.5 | 20.6 | 21.9 |
| Intercourse frequency <1 time/week, % | 20.8 | 21.5 | 21.8 | 22.2 | 23.0 | 20.5 | 22.8 | 23.4 | 21.6 | 21.7 |
| Doing something to improve chances of conception, % | 76.5 | 77.0 | 78.9 | 80.1 | 81.7 | 75.5 | 79.3 | 79.8 | 80.4 | 79.6 |
| History of STI, % | 17.8 | 14.5 | 12.9 | 11.5 | 10.9 | 16.1 | 13.3 | 12.8 | 11.6 | 12.5 |
| History of infertility, % | 14.2 | 9.7 | 9.2 | 6.3 | 5.9 | 10.7 | 8.0 | 8.0 | 8.0 | 10.0 |
| History of uterine leiomyomata, % | 2.4 | 2.0 | 2.6 | 2.3 | 2.2 | 3.1 | 1.9 | 2.4 | 1.9 | 2.1 |
| History of endometriosis, % | 3.3 | 3.1 | 3.5 | 3.5 | 2.1 | 2.8 | 2.9 | 2.8 | 3.6 | 3.6 |
| History of polycystic ovary syndrome, % | 11.4 | 9.3 | 8.9 | 7.9 | 7.9 | 10.0 | 9.1 | 8.3 | 8.8 | 8.9 |
| ≥1 visit to a primary care provider in the past year, % | 85.8 | 86.8 | 86.8 | 86.6 | 87.4 | 84.8 | 86.8 | 86.8 | 86.3 | 88.4 |
| High perceived stress (PSS score: ≥25), % | 11.5 | 10.2 | 9.0 | 8.6 | 7.1 | 11.7 | 7.5 | 8.4 | 8.5 | 10.2 |
| Severe depressive symptoms (MDI score: ≥30), % | 7.0 | 5.8 | 4.5 | 3.3 | 3.3 | 7.3 | 3.5 | 4.2 | 4.0 | 5.0 |
| <3 cycles of attempt time at enrollment, % | 60.4 | 64.4 | 67.1 | 68.7 | 71.1 | 62.1 | 68.7 | 68.3 | 67.5 | 65.9 |

Note: MDI = Major Depression Inventory; PRESTO = Pregnancy Study Online; PSS = Perceived Stress Scale; Q = quintile; STI = sexually transmitted infection (defined as chlamydia, genital herpes, or genital warts); ^a^Derived from the Index of Concentration at the Extremes; ^b^Standardized to the age distribution of the cohort at baseline; ^c^Includes American Indian, Alaskan Native, Indigenous, Middle Eastern or North African, some other race, or missing race.

**eTable 5.** Distribution of ICE metrics in PRESTO, overall and stratified by race/ethnicity

|  |  | **Mean (SD)** | **Median (IQR)** | **Minimum** | **Maximum** |
| --- | --- | --- | --- | --- | --- |
| Overall  (n=10,438) | ICE_education_ | 0.3 (0.3) | 0.3 (0.1, 0.5) | -0.6 | 1.0 |
|  | ICE_income_ | 0.1 (0.3) | 0.1 (0.0, 0.3) | -1.0 | 1.0 |
|  | ICE_white/black_ | 0.6 (0.3) | 0.7 (0.5, 0.9) | -1.0 | 1.0 |
|  | ICE_white/hispanic_ | 0.6 (0.3) | 0.7 (0.4, 0.8) | -1.0 | 1.0 |
|  | ICE_white/asian_ | 0.6 (0.3) | 0.7 (0.5, 0.9) | -0.7 | 1.0 |
|  | ICE_income + white/black_ | 0.2 (0.2) | 0.2 (0.1, 0.4) | -0.7 | 1.0 |
|  | ICE_income + white/hispanic_ | 0.1 (0.2) | 0.1 (-0.1, 0.3) | -1.0 | 1.0 |
|  | ICE_income + white/asian_ | 0.3 (0.2) | 0.2 (0.1, 0.4) | -0.3 | 1.0 |
| Non-Hispanic White  (n=8,707) | ICE_education_ | 0.3 (0.3) | 0.3 (0.1, 0.5) | -0.6 | 1.0 |
|  | ICE_income_ | 0.2 (0.3) | 0.2 (0.0, 0.3) | -1.0 | 1.0 |
|  | ICE_white/black_ | 0.6 (0.3) | 0.7 (0.5, 0.9) | -1.0 | 1.0 |
|  | ICE_white/hispanic_ | 0.6 (0.3) | 0.7 (0.5, 0.9) | -0.9 | 1.0 |
|  | ICE_white/asian_ | 0.7 (0.3) | 0.7 (0.5, 0.9) | -0.7 | 1.0 |
|  | ICE_income + white/black_ | 0.2 (0.2) | 0.2 (0.1, 0.4) | -0.7 | 1.0 |
|  | ICE_income + white/hispanic_ | 0.1 (0.2) | 0.1 (-0.1, 0.3) | -1.0 | 1.0 |
|  | ICE_income + white/asian_ | 0.3 (0.2) | 0.2 (0.1, 0.4) | -0.3 | 1.0 |
| Non-Hispanic Black  (n=343) | ICE_education_ | 0.2 (0.3) | 0.1 (0.0, 0.4) | -0.4 | 0.9 |
|  | ICE_income_ | 0.0 (0.3) | 0.0 (-0.2, 0.2) | -0.7 | 0.8 |
|  | ICE_white/black_ | 0.1 (0.6) | 0.2 (-0.4, 0.6) | -1.0 | 1.0 |
|  | ICE_white/hispanic_ | 0.3 (0.3) | 0.3 (0.1, 0.6) | -0.6 | 1.0 |
|  | ICE_white/asian_ | 0.4 (0.3) | 0.4 (0.1, 0.7) | 0.0 | 1.0 |
|  | ICE_income + white/black_ | 0.0 (0.2) | 0.0 (-0.1, 0.2) | -0.7 | 0.8 |
|  | ICE_income + white/hispanic_ | -0.1 (0.3) | -0.1 (-0.3, 0.0) | -0.7 | 0.7 |
|  | ICE_income + white/asian_ | 0.1 (0.1) | 0.1 (0.0, 0.2) | 0.0 | 0.8 |
| Hispanic  (n=772) | ICE_education_ | 0.2 (0.3) | 0.2 (0.1, 0.4) | -0.6 | 0.9 |
|  | ICE_income_ | 0.1 (0.3) | 0.1 (-0.1, 0.3) | -0.7 | 0.8 |
|  | ICE_white/black_ | 0.5 (0.3) | 0.5 (0.2, 0.7) | -0.9 | 1.0 |
|  | ICE_white/hispanic_ | 0.3 (0.5) | 0.5 (0.1, 0.7) | -1.0 | 1.0 |
|  | ICE_white/asian_ | 0.5 (0.3) | 0.6 (0.3, 0.7) | -0.4 | 1.0 |
|  | ICE_income + white/black_ | 0.2 (0.2) | 0.2 (0.1, 0.3) | -0.5 | 0.7 |
|  | ICE_income + white/hispanic_ | 0.0 (0.3) | 0.0 (-0.1, 0.2) | -0.7 | 0.7 |
|  | ICE_income + white/asian_ | 0.2 (0.2) | 0.2 (0.1, 0.3) | -0.2 | 0.7 |
| Non-Hispanic  Asian, Native Hawaiian, or Pacific Islander  (n=191) | ICE_education_ | 0.4 (0.3) | 0.4 (0.2, 0.6) | -0.4 | 1.0 |
|  | ICE_income_ | 0.2 (0.3) | 0.3 (0.0, 0.4) | -0.8 | 0.8 |
|  | ICE_white/black_ | 0.5 (0.3) | 0.6 (0.3, 0.8) | -0.8 | 1.0 |
|  | ICE_white/hispanic_ | 0.5 (0.3) | 0.5 (0.2, 0.7) | -0.5 | 1.0 |
|  | ICE_white/asian_ | 0.5 (0.3) | 0.5 (0.3, 0.7) | -0.5 | 1.0 |
|  | ICE_income + white/black_ | 0.3 (0.2) | 0.3 (0.1, 0.4) | -0.5 | 0.7 |
|  | ICE_income + white/hispanic_ | 0.1 (0.2) | 0.1 (0.0, 0.3) | -0.8 | 0.7 |
|  | ICE_income + white/asian_ | 0.3 (0.2) | 0.3 (0.1, 0.4) | -0.1 | 0.7 |
| Non-Hispanic Multiracial  (n=371) | ICE_education_ | 0.3 (0.2) | 0.2 (0.1, 0.5) | -0.3 | 0.9 |
|  | ICE_income_ | 0.1 (0.3) | 0.1 (-0.1, 0.3) | -0.6 | 0.8 |
|  | ICE_white/black_ | 0.5 (0.4) | 0.7 (0.4, 0.8) | -1.0 | 1.0 |
|  | ICE_white/hispanic_ | 0.5 (0.3) | 0.6 (0.3, 0.8) | -0.5 | 1.0 |
|  | ICE_white/asian_ | 0.6 (0.3) | 0.7 (0.4, 0.8) | -0.4 | 1.0 |
|  | ICE_income + white/black_ | 0.2 (0.2) | 0.2 (0.1, 0.3) | -0.6 | 0.8 |
|  | ICE_income + white/hispanic_ | 0.0 (0.2) | 0.1 (-0.1, 0.2) | -0.6 | 0.7 |
|  | ICE_income + white/asian_ | 0.2 (0.1) | 0.2 (0.1, 0.3) | -0.2 | 0.8 |
| Non-Hispanic Other race^a^  (n=54) | ICE_education_ | 0.3 (0.3) | 0.3 (0.1, 0.5) | -0.1 | 0.9 |
|  | ICE_income_ | 0.2 (0.3) | 0.2 (-0.1, 0.4) | -0.4 | 0.7 |
|  | ICE_white/black_ | 0.6 (0.4) | 0.7 (0.4, 0.8) | -0.9 | 1.0 |
|  | ICE_white/hispanic_ | 0.5 (0.3) | 0.6 (0.3, 0.8) | -0.5 | 1.0 |
|  | ICE_white/asian_ | 0.6 (0.3) | 0.6 (0.4, 0.8) | 0.0 | 1.0 |
|  | ICE_income + white/black_ | 0.2 (0.2) | 0.2 (0.1, 0.4) | -0.3 | 0.7 |
|  | ICE_income + white/hispanic_ | 0.1 (0.3) | 0.1 (-0.1, 0.3) | -0.4 | 0.6 |
|  | ICE_income + white/asian_ | 0.3 (0.2) | 0.3 (0.1, 0.4) | 0.0 | 0.7 |

Note: ICE = Index of Concentration at the Extremes; IQR = interquartile range; PRESTO = Pregnancy Study Online; SD = standard deviation; ^a^Includes American Indian, Alaskan Native, Indigenous, Middle Eastern or North African, some other race, or missing race.

**eTable 6.** Correlation matrix^a^ of ICE metrics, PRESTO 2013-2022

|  | **ICE_education_** | **ICE_income_** | **ICE_white/black_** | **ICE_white/hispanic_** | **ICE_white/asian_** | **ICE_income + white/black_** | **ICE_income + white/hispanic_** | **ICE_income + white/asian_** |
| --- | --- | --- | --- | --- | --- | --- | --- | --- |
| ICE_education_ | 1.00 |  |  |  |  |  |  |  |
| ICE_income_ | 0.70 | 1.00 |  |  |  |  |  |  |
| ICE_white/black_ | 0.14 | 0.20 | 1.00 |  |  |  |  |  |
| ICE_white/hispanic_ | 0.16 | 0.18 | 0.94 | 1.00 |  |  |  |  |
| ICE_white/asian_ | 0.04 | 0.10 | 0.96 | 0.96 | 1.00 |  |  |  |
| ICE_income + white/black_ | 0.67 | 0.89 | 0.51 | 0.46 | 0.40 | 1.00 |  |  |
| ICE_income + white/hispanic_ | 0.68 | 0.96 | 0.38 | 0.35 | 0.29 | 0.96 | 1.00 |  |
| ICE_income + white/asian_ | 0.68 | 0.89 | 0.47 | 0.45 | 0.39 | 0.98 | 0.96 | 1.00 |

Note: ICE = Index of Concentration at the Extremes; PRESTO = Pregnancy Study Online; ^a^Spearman correlation (range: -1.00 to 1.00).

**eTable 7.** Associations between economic, racial, and racialized economic segregation with fecundability, PRESTO 2013-2022

|  | **Pregnancies** | **Cycles** | **Unadjusted FR (95% CI)** | **Adjusted FR**  **(95% CI)^a^** | **Adjusted FR**  **(95% CI)^b^** | **Adjusted FR**  **(95% CI)^c^** |
| --- | --- | --- | --- | --- | --- | --- |
| **ICE_income_** |  |  |  |  |  |  |
| Q1 (most disadvantaged) | 912 | 7,616 | 0.77 (0.71-0.83) | 0.80 (0.74-0.87) | 0.83 (0.76-0.89) | 0.91 (0.83-0.98) |
| Q2 | 1,130 | 8,239 | 0.84 (0.78-0.90) | 0.86 (0.80-0.92) | 0.87 (0.81-0.94) | 0.93 (0.86-1.00) |
| Q3 | 1,289 | 8,915 | 0.88 (0.82-0.94) | 0.89 (0.83-0.95) | 0.91 (0.84-0.97) | 0.94 (0.88-1.01) |
| Q4 | 1,401 | 8,934 | 0.93 (0.87-1.00) | 0.94 (0.88-1.00) | 0.94 (0.88-1.01) | 0.96 (0.90-1.03) |
| Q5 (most privileged) | 1,506 | 8,871 | 1.00 (Reference) | 1.00 (Reference) | 1.00 (Reference) | 1.00 (Reference) |
| **ICE_white/black_** |  |  |  |  |  |  |
| Q1 (most disadvantaged) | 1,043 | 7,863 | 0.90 (0.84-0.97) | 0.93 (0.85-1.00) | 0.94 (0.87-1.02) | 0.94 (0.87-1.02) |
| Q2 | 1,288 | 8,858 | 0.96 (0.89-1.03) | 0.97 (0.90-1.05) | 0.98 (0.91-1.05) | 0.95 (0.88-1.02) |
| Q3 | 1,328 | 9,002 | 0.98 (0.92-1.06) | 0.99 (0.92-1.06) | 1.00 (0.92-1.07) | 0.97 (0.90-1.04) |
| Q4 | 1,337 | 8,630 | 1.02 (0.95-1.10) | 1.02 (0.95-1.10) | 1.02 (0.95-1.09) | 0.99 (0.92-1.06) |
| Q5 (most privileged) | 1,242 | 8,222 | 1.00 (Reference) | 1.00 (Reference) | 1.00 (Reference) | 1.00 (Reference) |
| **ICE_income + white/black_** |  |  |  |  |  |  |
| Q1 (most disadvantaged) | 937 | 7,668 | 0.76 (0.71-0.82) | 0.79 (0.73-0.86) | 0.82 (0.76-0.89) | 0.89 (0.82-0.97) |
| Q2 | 1,149 | 8,461 | 0.82 (0.76-0.88) | 0.84 (0.78-0.90) | 0.86 (0.80-0.92) | 0.91 (0.84-0.98) |
| Q3 | 1,204 | 8,633 | 0.83 (0.78-0.89) | 0.84 (0.78-0.90) | 0.86 (0.80-0.92) | 0.89 (0.83-0.96) |
| Q4 | 1,420 | 9,058 | 0.92 (0.86-0.98) | 0.92 (0.86-0.99) | 0.93 (0.87-1.00) | 0.95 (0.89-1.01) |
| Q5 (most privileged) | 1,528 | 8,755 | 1.00 (Reference) | 1.00 (Reference) | 1.00 (Reference) | 1.00 (Reference) |
| **ICE_education_** |  |  |  |  |  |  |
| Q1 (most disadvantaged) | 855 | 7,074 | 0.77 (0.71-0.83) | 0.78 (0.72-0.85) | 0.81 (0.75-0.88) | 0.90 (0.83-0.98) |
| Q2 | 1,068 | 8,203 | 0.81 (0.75-0.87) | 0.80 (0.75-0.86) | 0.83 (0.77-0.89) | 0.90 (0.83-0.97) |
| Q3 | 1,293 | 8,958 | 0.88 (0.82-0.94) | 0.87 (0.82-0.94) | 0.89 (0.83-0.95) | 0.94 (0.88-1.01) |
| Q4 | 1,423 | 9,024 | 0.95 (0.89-1.02) | 0.95 (0.89-1.01) | 0.95 (0.89-1.02) | 0.98 (0.91-1.04) |
| Q5 (most privileged) | 1,599 | 9,316 | 1.00 (Reference) | 1.00 (Reference) | 1.00 (Reference) | 1.00 (Reference) |
| **ICE_white/hispanic_** |  |  |  |  |  |  |
| Q1 (most disadvantaged) | 1,067 | 8,014 | 0.91 (0.84-0.98) | 0.93 (0.86-1.01) | 0.95 (0.88-1.03) | 0.95 (0.88-1.04) |
| Q2 | 1,287 | 8,749 | 0.99 (0.92-1.06) | 1.00 (0.93-1.08) | 1.00 (0.93-1.08) | 0.99 (0.91-1.06) |
| Q3 | 1,327 | 8,904 | 1.00 (0.93-1.07) | 1.01 (0.94-1.08) | 1.01 (0.94-1.09) | 0.98 (0.91-1.06) |
| Q4 | 1,311 | 8,539 | 1.02 (0.95-1.09) | 1.02 (0.95-1.10) | 1.02 (0.95-1.10) | 1.00 (0.93-1.08) |
| Q5 (most privileged) | 1,246 | 8,369 | 1.00 (Reference) | 1.00 (Reference) | 1.00 (Reference) | 1.00 (Reference) |
| **ICE_white/asian_** |  |  |  |  |  |  |
| Q1 (most disadvantaged) | 1,136 | 8,366 | 0.94 (0.87-1.01) | 0.96 (0.89-1.04) | 0.98 (0.90-1.06) | 0.97 (0.89-1.05) |
| Q2 | 1,263 | 8,564 | 1.00 (0.93-1.08) | 1.02 (0.95-1.10) | 1.03 (0.95-1.11) | 1.00 (0.93-1.08) |
| Q3 | 1,314 | 8,903 | 1.01 (0.93-1.08) | 1.02 (0.94-1.10) | 1.02 (0.95-1.10) | 0.99 (0.92-1.07) |
| Q4 | 1,319 | 8,544 | 1.05 (0.98-1.13) | 1.06 (0.98-1.14) | 1.05 (0.98-1.13) | 1.03 (0.96-1.11) |
| Q5 (most privileged) | 1,206 | 8,198 | 1.00 (Reference) | 1.00 (Reference) | 1.00 (Reference) | 1.00 (Reference) |
| **ICE_income + white/hispanic_** |  |  |  |  |  |  |
| Q1 (most disadvantaged) | 923 | 7,652 | 0.75 (0.70-0.81) | 0.79 (0.73-0.85) | 0.81 (0.75-0.88) | 0.89 (0.82-0.96) |
| Q2 | 1,105 | 8,126 | 0.82 (0.76-0.88) | 0.84 (0.78-0.90) | 0.86 (0.80-0.92) | 0.91 (0.84-0.98) |
| Q3 | 1,291 | 8,998 | 0.86 (0.80-0.92) | 0.87 (0.81-0.93) | 0.88 (0.82-0.94) | 0.92 (0.85-0.98) |
| Q4 | 1,393 | 9,022 | 0.90 (0.85-0.97) | 0.91 (0.85-0.97) | 0.92 (0.86-0.98) | 0.93 (0.87-1.00) |
| Q5 (most privileged) | 1,526 | 8,777 | 1.00 (Reference) | 1.00 (Reference) | 1.00 (Reference) | 1.00 (Reference) |
| **ICE_income + white/asian_** |  |  |  |  |  |  |
| Q1 (most disadvantaged) | 958 | 7,821 | 0.77 (0.71-0.83) | 0.80 (0.74-0.86) | 0.83 (0.76-0.89) | 0.90 (0.83-0.98) |
| Q2 | 1,120 | 8,404 | 0.81 (0.75-0.87) | 0.83 (0.77-0.90) | 0.85 (0.79-0.92) | 0.90 (0.84-0.97) |
| Q3 | 1,211 | 8,581 | 0.85 (0.79-0.91) | 0.85 (0.80-0.92) | 0.87 (0.81-0.93) | 0.90 (0.84-0.97) |
| Q4 | 1,428 | 8,976 | 0.94 (0.88-1.00) | 0.94 (0.88-1.01) | 0.95 (0.89-1.02) | 0.97 (0.91-1.04) |
| Q5 (most privileged) | 1,521 | 8,793 | 1.00 (Reference) | 1.00 (Reference) | 1.00 (Reference) | 1.00 (Reference) |

Note: CI = confidence interval; FR = fecundability ratio; PRESTO = Pregnancy Study Online; ^a^Adjusted for age, calendar year of enrollment, and geographic region of residence; ^b^Additionally adjusted for multivitamin use, last method of contraception, and intercourse frequency; ^c^Additionally adjusted for participants’ race/ethnicity, educational attainment, and household income

**eFigure 3.** Associations between other metrics of economic, racial, and racialized economic segregation with fecundability, PRESTO 2013-2022


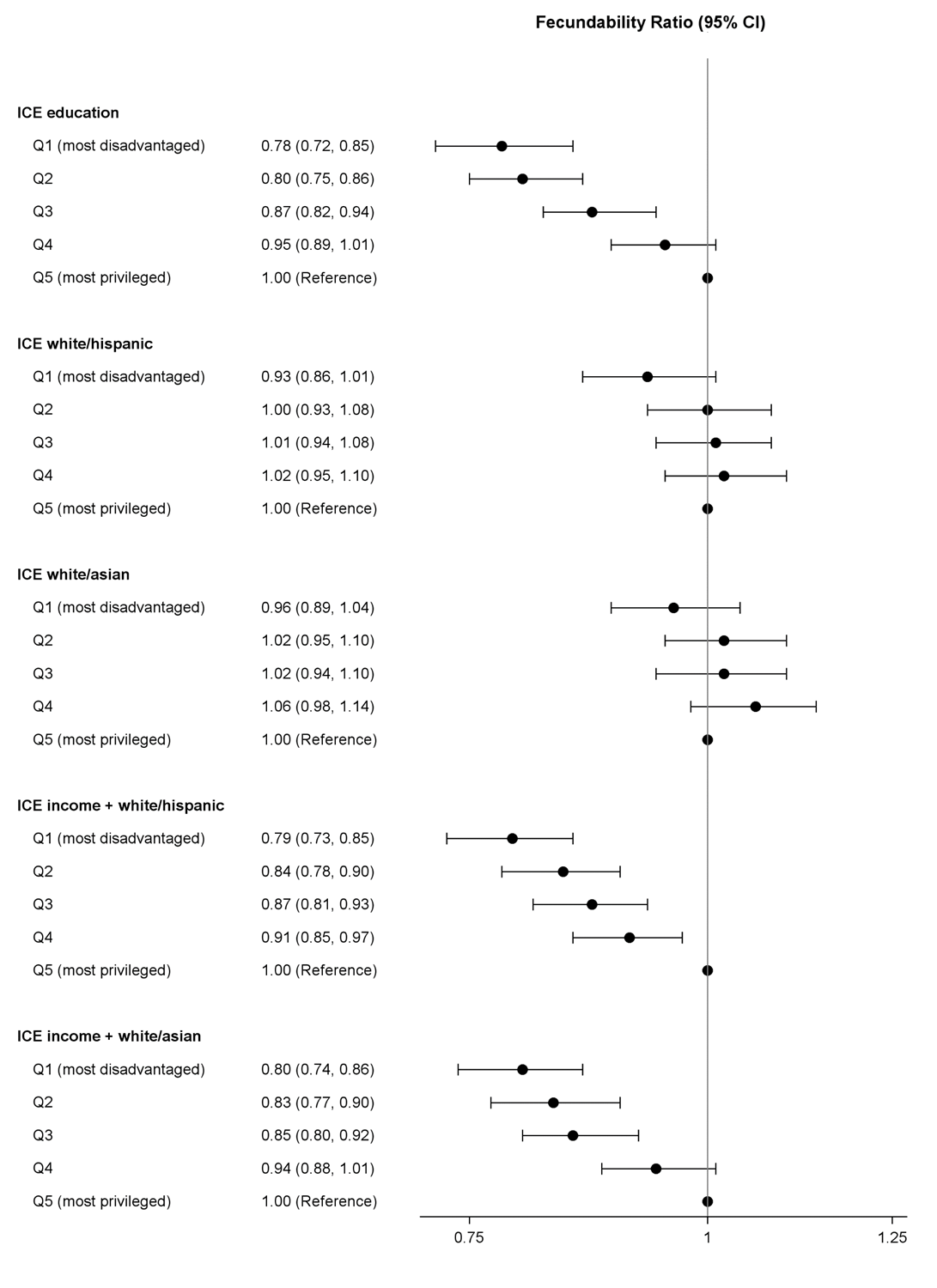


Note: CI = confidence interval; ICE = Index of Concentration at the Extremes; PRESTO = Pregnancy Study Online; Adjusted for age, calendar year of enrollment, and geographic region of residence

**eFigure 4.** Restricted cubic splines for associations between other metrics of economic, racial, and racialized economic segregation with fecundability, PRESTO 2013-2022

| **ICE_education_** | |
| --- | --- |
| 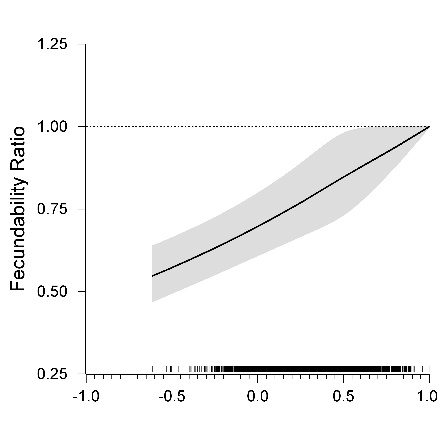 | |
| **ICE_white/hispanic_** | **ICE_white/asian_** |
| 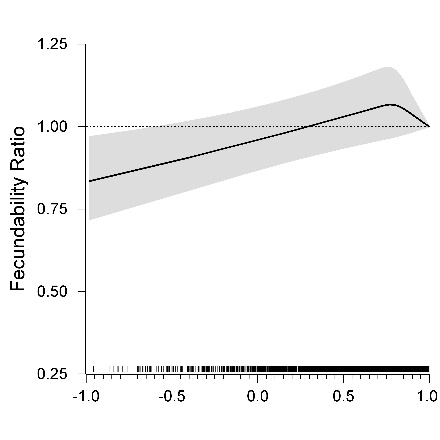 | 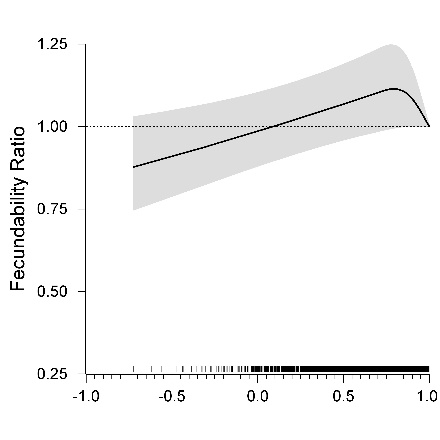 |
| **ICE_income + white/hispanic_** | **ICE_income + white/asian_** |
| 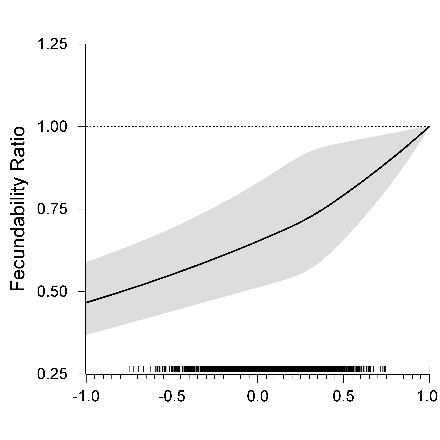 | 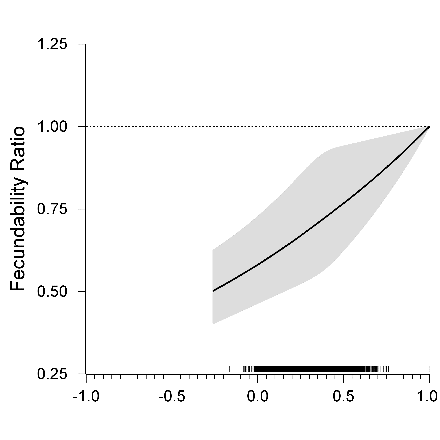 |

Note: PRESTO = Pregnancy Study Online; Graphs are plots of restricted cubic splines. Knots are located at the 50th, 75th, and 95th percentiles (ICE_education_: 0.3, 0.5, 0.7; ICE_white/hispanic_: 0.7, 0.8, 0.9; ICE_white/asian_: 0.7, 0.9, 1.0; ICE_income + white/hispanic_: 0.1, 0.3, 0.5; ICE_income + white/asian_: 0.2, 0.4, 0.5). The reference level is the maximum value of the exposure (score = 1.0). The black solid line indicates the fecundability ratio (FR) and the shaded gray area is the 95% confidence interval (CI); FRs are adjusted for age, calendar year of enrollment, and geographic region of residence. **If the spline appears truncated, this signifies the minimum value of exposure is greater than -1.0.**

**eTable 8.** Associations between economic, racial, and racialized economic segregation with fecundability stratified by pregnancy attempt time at enrollment, PRESTO 2013-2022

|  | **<3 Cycles of Pregnancy Attempt Time**  **at Enrollment** | | | **3-6 Cycles of Pregnancy Attempt Time**  **at Enrollment** | | |
| --- | --- | --- | --- | --- | --- | --- |
|  | (n=6,955) | | | (n=3,483) | | |
|  | **Pregnancies** | **Cycles** | **Adjusted FR**  **(95% CI)** | **Pregnancies** | **Cycles** | **Adjusted FR**  **(95% CI)** |
| **ICE_income_** |  |  |  |  |  |  |
| Q1 (most disadvantaged) | 649 | 4,667 | 0.82 (0.75-0.90) | 263 | 2,949 | 0.76 (0.64-0.89) |
| Q2 | 821 | 5,529 | 0.84 (0.77-0.91) | 309 | 2,710 | 0.92 (0.79-1.08) |
| Q3 | 960 | 6,203 | 0.87 (0.80-0.94) | 329 | 2,712 | 0.96 (0.83-1.12) |
| Q4 | 1,059 | 6,328 | 0.91 (0.85-0.99) | 342 | 2,606 | 1.04 (0.90-1.21) |
| Q5 (most privileged) | 1,179 | 6,321 | 1.00 (Reference) | 327 | 2,550 | 1.00 (Reference) |
| **ICE_white/black_** |  |  |  |  |  |  |
| Q1 (most disadvantaged) | 769 | 4,957 | 0.97 (0.88-1.07) | 274 | 2,906 | 0.81 (0.69-0.95) |
| Q2 | 983 | 6,253 | 0.97 (0.89-1.06) | 305 | 2,605 | 0.96 (0.82-1.12) |
| Q3 | 986 | 6,327 | 0.97 (0.89-1.05) | 342 | 2,675 | 1.05 (0.90-1.22) |
| Q4 | 1,013 | 5,959 | 1.03 (0.95-1.12) | 324 | 2,671 | 0.99 (0.85-1.15) |
| Q5 (most privileged) | 917 | 5,552 | 1.00 (Reference) | 325 | 2,670 | 1.00 (Reference) |
| **ICE_income + white/black_** |  |  |  |  |  |  |
| Q1 (most disadvantaged) | 680 | 4,690 | 0.84 (0.76-0.91) | 257 | 2,978 | 0.69 (0.59-0.81) |
| Q2 | 826 | 5,599 | 0.83 (0.76-0.90) | 323 | 2,862 | 0.88 (0.75-1.02) |
| Q3 | 907 | 6,075 | 0.83 (0.77-0.90) | 297 | 2,558 | 0.87 (0.74-1.01) |
| Q4 | 1,062 | 6,418 | 0.90 (0.83-0.97) | 358 | 2,640 | 1.01 (0.87-1.16) |
| Q5 (most privileged) | 1,193 | 6,266 | 1.00 (Reference) | 335 | 2,489 | 1.00 (Reference) |
| **ICE_education_** |  |  |  |  |  |  |
| Q1 (most disadvantaged) | 588 | 4,273 | 0.81 (0.74-0.89) | 267 | 2,801 | 0.71 (0.61-0.84) |
| Q2 | 777 | 5,363 | 0.82 (0.75-0.89) | 291 | 2,840 | 0.76 (0.65-0.89) |
| Q3 | 949 | 6,181 | 0.86 (0.80-0.93) | 344 | 2,777 | 0.89 (0.77-1.03) |
| Q4 | 1,085 | 6,250 | 0.96 (0.89-1.04) | 338 | 2,774 | 0.89 (0.77-1.03) |
| Q5 (most privileged) | 1,269 | 6,981 | 1.00 (Reference) | 330 | 2,335 | 1.00 (Reference) |
| **ICE_white/hispanic_** |  |  |  |  |  |  |
| Q1 (most disadvantaged) | 789 | 5,140 | 0.97 (0.88-1.06) | 278 | 2,874 | 0.83 (0.71-0.98) |
| Q2 | 971 | 6,030 | 1.00 (0.92-1.09) | 316 | 2,719 | 1.00 (0.85-1.16) |
| Q3 | 999 | 6,303 | 0.99 (0.91-1.08) | 328 | 2,601 | 1.06 (0.91-1.24) |
| Q4 | 990 | 5,959 | 1.02 (0.94-1.11) | 321 | 2,580 | 1.03 (0.89-1.20) |
| Q5 (most privileged) | 919 | 5,616 | 1.00 (Reference) | 327 | 2,753 | 1.00 (Reference) |
| **ICE_white/asian_** |  |  |  |  |  |  |
| Q1 (most disadvantaged) | 843 | 5,458 | 0.99 (0.90-1.08) | 293 | 2,908 | 0.87 (0.75-1.02) |
| Q2 | 969 | 5,959 | 1.04 (0.96-1.14) | 294 | 2,605 | 0.97 (0.83-1.13) |
| Q3 | 1,002 | 6,321 | 1.02 (0.93-1.11) | 312 | 2,582 | 1.01 (0.87-1.18) |
| Q4 | 961 | 5,758 | 1.05 (0.97-1.15) | 358 | 2,786 | 1.07 (0.92-1.24) |
| Q5 (most privileged) | 893 | 5,552 | 1.00 (Reference) | 313 | 2,646 | 1.00 (Reference) |
| **ICE_income + white/hispanic_** |  |  |  |  |  |  |
| Q1 (most disadvantaged) | 659 | 4,691 | 0.81 (0.74-0.89) | 264 | 2,961 | 0.73 (0.62-0.85) |
| Q2 | 797 | 5,403 | 0.83 (0.76-0.90) | 308 | 2,723 | 0.87 (0.75-1.02) |
| Q3 | 969 | 6,261 | 0.86 (0.79-0.93) | 322 | 2,737 | 0.91 (0.78-1.06) |
| Q4 | 1,048 | 6,401 | 0.88 (0.82-0.95) | 345 | 2,621 | 1.01 (0.87-1.16) |
| Q5 (most privileged) | 1,195 | 6,292 | 1.00 (Reference) | 331 | 2,485 | 1.00 (Reference) |
| **ICE_income + white/asian_** |  |  |  |  |  |  |
| Q1 (most disadvantaged) | 692 | 4,826 | 0.83 (0.76-0.91) | 266 | 2,995 | 0.72 (0.61-0.85) |
| Q2 | 815 | 5,578 | 0.83 (0.76-0.90) | 305 | 2,826 | 0.85 (0.73-0.99) |
| Q3 | 910 | 6,035 | 0.84 (0.78-0.91) | 301 | 2,546 | 0.90 (0.77-1.05) |
| Q4 | 1,060 | 6,313 | 0.92 (0.85-0.99) | 368 | 2,663 | 1.03 (0.90-1.19) |
| Q5 (most privileged) | 1,191 | 6,296 | 1.00 (Reference) | 330 | 2,497 | 1.00 (Reference) |

Note: CI = confidence interval; FR = fecundability ratio; PRESTO = Pregnancy Study Online; Adjusted for age, calendar year of enrollment, and geographic region of residence

**eTable 9.** Associations between economic, racial, and racialized economic segregation with fecundability stratified by parity, PRESTO 2013-2022

|  | **Nulliparous** | | | **Parous** | | |
| --- | --- | --- | --- | --- | --- | --- |
|  | (n=6,860) | | | (n=3,578) | | |
|  | **Pregnancies** | **Cycles** | **Adjusted FR**  **(95% CI)** | **Pregnancies** | **Cycles** | **Adjusted FR**  **(95% CI)** |
| **ICE_income_** |  |  |  |  |  |  |
| Q1 (most disadvantaged) | 566 | 4,965 | 0.81 (0.74-0.90) | 346 | 2,651 | 0.75 (0.66-0.86) |
| Q2 | 741 | 5,699 | 0.87 (0.80-0.96) | 389 | 2,540 | 0.82 (0.73-0.93) |
| Q3 | 848 | 6,177 | 0.92 (0.84-1.00) | 441 | 2,738 | 0.84 (0.75-0.95) |
| Q4 | 910 | 6,216 | 0.95 (0.88-1.04) | 491 | 2,718 | 0.91 (0.81-1.02) |
| Q5 (most privileged) | 975 | 6,253 | 1.00 (Reference) | 531 | 2,618 | 1.00 (Reference) |
| **ICE_white/black_** |  |  |  |  |  |  |
| Q1 (most disadvantaged) | 703 | 5,552 | 0.91 (0.82-1.01) | 340 | 2,311 | 1.00 (0.88-1.15) |
| Q2 | 876 | 6,429 | 0.95 (0.86-1.04) | 412 | 2,429 | 1.06 (0.93-1.21) |
| Q3 | 861 | 6,304 | 0.95 (0.87-1.04) | 467 | 2,698 | 1.08 (0.96-1.22) |
| Q4 | 823 | 5,712 | 0.99 (0.90-1.09) | 514 | 2,918 | 1.09 (0.97-1.23) |
| Q5 (most privileged) | 777 | 5,313 | 1.00 (Reference) | 465 | 2,909 | 1.00 (Reference) |
| **ICE_income + white/black_** |  |  |  |  |  |  |
| Q1 (most disadvantaged) | 634 | 5,244 | 0.84 (0.76-0.92) | 303 | 2,424 | 0.72 (0.63-0.82) |
| Q2 | 727 | 5,775 | 0.84 (0.77-0.92) | 422 | 2,686 | 0.83 (0.73-0.93) |
| Q3 | 751 | 5,830 | 0.85 (0.78-0.93) | 453 | 2,803 | 0.82 (0.73-0.92) |
| Q4 | 953 | 6,370 | 0.95 (0.88-1.03) | 467 | 2,688 | 0.88 (0.79-0.99) |
| Q5 (most privileged) | 975 | 6,091 | 1.00 (Reference) | 553 | 2,664 | 1.00 (Reference) |
| **ICE_education_** |  |  |  |  |  |  |
| Q1 (most disadvantaged) | 492 | 4,227 | 0.81 (0.73-0.90) | 363 | 2,847 | 0.65 (0.57-0.74) |
| Q2 | 659 | 5,312 | 0.82 (0.75-0.90) | 409 | 2,891 | 0.69 (0.61-0.78) |
| Q3 | 852 | 6,363 | 0.88 (0.81-0.95) | 441 | 2,595 | 0.82 (0.72-0.92) |
| Q4 | 924 | 6,308 | 0.96 (0.88-1.04) | 499 | 2,716 | 0.86 (0.77-0.96) |
| Q5 (most privileged) | 1,113 | 7,100 | 1.00 (Reference) | 486 | 2,216 | 1.00 (Reference) |
| **ICE_white/hispanic_** |  |  |  |  |  |  |
| Q1 (most disadvantaged) | 718 | 5,601 | 0.91 (0.82-1.01) | 349 | 2,413 | 1.01 (0.88-1.16) |
| Q2 | 867 | 6,280 | 0.97 (0.88-1.07) | 420 | 2,469 | 1.10 (0.97-1.25) |
| Q3 | 864 | 6,314 | 0.96 (0.87-1.05) | 463 | 2,590 | 1.15 (1.01-1.30) |
| Q4 | 795 | 5,672 | 0.97 (0.88-1.06) | 516 | 2,867 | 1.14 (1.01-1.28) |
| Q5 (most privileged) | 796 | 5,443 | 1.00 (Reference) | 450 | 2,926 | 1.00 (Reference) |
| **ICE_white/asian_** |  |  |  |  |  |  |
| Q1 (most disadvantaged) | 784 | 6,117 | 0.94 (0.85-1.04) | 352 | 2,249 | 1.06 (0.93-1.21) |
| Q2 | 847 | 6,036 | 1.02 (0.93-1.13) | 416 | 2,528 | 1.06 (0.93-1.20) |
| Q3 | 850 | 6,302 | 0.98 (0.89-1.07) | 464 | 2,601 | 1.14 (1.01-1.29) |
| Q4 | 813 | 5,596 | 1.04 (0.95-1.15) | 506 | 2,948 | 1.09 (0.97-1.23) |
| Q5 (most privileged) | 746 | 5,259 | 1.00 (Reference) | 460 | 2,939 | 1.00 (Reference) |
| **ICE_income + white/hispanic_** |  |  |  |  |  |  |
| Q1 (most disadvantaged) | 588 | 5,089 | 0.80 (0.72-0.88) | 335 | 2,563 | 0.76 (0.66-0.86) |
| Q2 | 715 | 5,644 | 0.84 (0.76-0.92) | 390 | 2,482 | 0.85 (0.75-0.96) |
| Q3 | 852 | 6,200 | 0.90 (0.82-0.98) | 439 | 2,798 | 0.83 (0.73-0.93) |
| Q4 | 909 | 6,311 | 0.91 (0.84-0.99) | 484 | 2,711 | 0.91 (0.82-1.02) |
| Q5 (most privileged) | 976 | 6,066 | 1.00 (Reference) | 550 | 2,711 | 1.00 (Reference) |
| **ICE_income + white/asian_** |  |  |  |  |  |  |
| Q1 (most disadvantaged) | 638 | 5,265 | 0.85 (0.77-0.93) | 320 | 2,556 | 0.71 (0.63-0.82) |
| Q2 | 710 | 5,792 | 0.84 (0.76-0.92) | 410 | 2,612 | 0.82 (0.73-0.93) |
| Q3 | 768 | 5,846 | 0.87 (0.79-0.95) | 443 | 2,735 | 0.83 (0.74-0.93) |
| Q4 | 957 | 6,290 | 0.98 (0.90-1.06) | 471 | 2,686 | 0.89 (0.80-1.00) |
| Q5 (most privileged) | 967 | 6,117 | 1.00 (Reference) | 554 | 2,676 | 1.00 (Reference) |

Note: CI = confidence interval; FR = fecundability ratio; PRESTO = Pregnancy Study Online; Adjusted for age, calendar year of enrollment, and geographic region of residence

**eTable 10.** Associations between economic, racial, and racialized economic segregation with fecundability stratified by infertility history, PRESTO 2013-2022

|  | **History of Infertility** | | | **No History of Infertility** | | |
| --- | --- | --- | --- | --- | --- | --- |
|  | (n=926) | | | (n=9,512) | | |
|  | **Pregnancies** | **Cycles** | **Adjusted FR**  **(95% CI)** | **Pregnancies** | **Cycles** | **Adjusted FR**  **(95% CI)** |
| **ICE_income_** |  |  |  |  |  |  |
| Q1 (most disadvantaged) | 64 | 909 | 0.78 (0.54-1.12) | 848 | 6,707 | 0.82 (0.76-0.89) |
| Q2 | 61 | 749 | 0.91 (0.64-1.30) | 1,069 | 7,490 | 0.86 (0.80-0.93) |
| Q3 | 59 | 840 | 0.73 (0.51-1.05) | 1,230 | 8,075 | 0.91 (0.85-0.98) |
| Q4 | 59 | 528 | 1.09 (0.77-1.54) | 1,342 | 8,406 | 0.93 (0.87-1.00) |
| Q5 (most privileged) | 63 | 615 | 1.00 (Reference) | 1,443 | 8,256 | 1.00 (Reference) |
| **ICE_white/black_** |  |  |  |  |  |  |
| Q1 (most disadvantaged) | 58 | 686 | 1.02 (0.70-1.49) | 985 | 7,177 | 0.91 (0.84-0.99) |
| Q2 | 44 | 706 | 0.82 (0.55-1.23) | 1,244 | 8,152 | 0.96 (0.89-1.04) |
| Q3 | 74 | 723 | 1.33 (0.96-1.86) | 1,254 | 8,279 | 0.96 (0.89-1.04) |
| Q4 | 61 | 628 | 1.22 (0.86-1.72) | 1,276 | 8,002 | 1.00 (0.93-1.08) |
| Q5 (most privileged) | 69 | 898 | 1.00 (Reference) | 1,173 | 7,324 | 1.00 (Reference) |
| **ICE_income + white/black_** |  |  |  |  |  |  |
| Q1 (most disadvantaged) | 59 | 875 | 0.62 (0.43-0.91) | 878 | 6,793 | 0.82 (0.76-0.89) |
| Q2 | 74 | 820 | 0.92 (0.65-1.29) | 1,075 | 7,641 | 0.85 (0.78-0.91) |
| Q3 | 48 | 711 | 0.61 (0.42-0.88) | 1,156 | 7,922 | 0.86 (0.80-0.92) |
| Q4 | 58 | 683 | 0.77 (0.55-1.10) | 1,362 | 8,375 | 0.93 (0.87-1.00) |
| Q5 (most privileged) | 67 | 552 | 1.00 (Reference) | 1,461 | 8,203 | 1.00 (Reference) |
| **ICE_education_** |  |  |  |  |  |  |
| Q1 (most disadvantaged) | 64 | 939 | 0.70 (0.49-1.01) | 791 | 6,135 | 0.81 (0.75-0.88) |
| Q2 | 72 | 884 | 0.87 (0.62-1.23) | 996 | 7,319 | 0.81 (0.75-0.88) |
| Q3 | 60 | 704 | 0.87 (0.60-1.25) | 1,233 | 8,254 | 0.88 (0.82-0.95) |
| Q4 | 50 | 584 | 0.86 (0.59-1.25) | 1,373 | 8,440 | 0.95 (0.89-1.02) |
| Q5 (most privileged) | 60 | 530 | 1.00 (Reference) | 1,539 | 8,786 | 1.00 (Reference) |
| **ICE_white/hispanic_** |  |  |  |  |  |  |
| Q1 (most disadvantaged) | 46 | 630 | 0.87 (0.58-1.29) | 1,021 | 7,384 | 0.92 (0.85-1.00) |
| Q2 | 60 | 830 | 0.88 (0.62-1.25) | 1,227 | 7,919 | 1.00 (0.93-1.08) |
| Q3 | 66 | 646 | 1.31 (0.94-1.83) | 1,261 | 8,258 | 0.98 (0.91-1.06) |
| Q4 | 61 | 599 | 1.15 (0.82-1.62) | 1,250 | 7,940 | 1.00 (0.93-1.08) |
| Q5 (most privileged) | 73 | 936 | 1.00 (Reference) | 1,173 | 7,433 | 1.00 (Reference) |
| **ICE_white/asian_** |  |  |  |  |  |  |
| Q1 (most disadvantaged) | 51 | 629 | 0.95 (0.65-1.39) | 1,085 | 7,737 | 0.94 (0.87-1.02) |
| Q2 | 52 | 795 | 0.77 (0.53-1.12) | 1,211 | 7,769 | 1.03 (0.95-1.11) |
| Q3 | 66 | 668 | 1.32 (0.95-1.83) | 1,248 | 8,235 | 0.99 (0.92-1.07) |
| Q4 | 62 | 608 | 1.21 (0.87-1.69) | 1,257 | 7,936 | 1.03 (0.96-1.11) |
| Q5 (most privileged) | 75 | 941 | 1.00 (Reference) | 1,131 | 7,257 | 1.00 (Reference) |
| **ICE_income + white/hispanic_** |  |  |  |  |  |  |
| Q1 (most disadvantaged) | 65 | 900 | 0.69 (0.48-1.00) | 858 | 6,752 | 0.81 (0.75-0.88) |
| Q2 | 64 | 759 | 0.86 (0.60-1.22) | 1,041 | 7,367 | 0.85 (0.79-0.91) |
| Q3 | 53 | 781 | 0.65 (0.45-0.94) | 1,238 | 8,217 | 0.89 (0.83-0.96) |
| Q4 | 60 | 643 | 0.82 (0.58-1.16) | 1,333 | 8,379 | 0.91 (0.85-0.98) |
| Q5 (most privileged) | 64 | 558 | 1.00 (Reference) | 1,462 | 8,219 | 1.00 (Reference) |
| **ICE_income + white/asian_** |  |  |  |  |  |  |
| Q1 (most disadvantaged) | 68 | 855 | 0.75 (0.53-1.08) | 890 | 6,966 | 0.81 (0.75-0.88) |
| Q2 | 55 | 838 | 0.65 (0.45-0.94) | 1,065 | 7,566 | 0.86 (0.79-0.92) |
| Q3 | 53 | 728 | 0.69 (0.48-1.00) | 1,158 | 7,853 | 0.87 (0.81-0.93) |
| Q4 | 64 | 665 | 0.86 (0.61-1.20) | 1,364 | 8,311 | 0.95 (0.89-1.02) |
| Q5 (most privileged) | 66 | 555 | 1.00 (Reference) | 1,455 | 8,238 | 1.00 (Reference) |

Note: CI = confidence interval; FR = fecundability ratio; PRESTO = Pregnancy Study Online; Adjusted for age, calendar year of enrollment, and geographic region of residence

**eTable 11.** Associations between economic, racial, and racialized economic segregation with fecundability among nulligravid participants with <3 cycles of pregnancy attempt time at enrollment, overall and further restricted to participants without a history of infertility, PRESTO 2013-2022

|  | **Nulligravid with <3 Cycles**  **of Attempt Time at Enrollment** | | | **Nulligravid with <3 Cycles**  **of Attempt Time at Enrollment**  **and No History of Infertility** | | |
| --- | --- | --- | --- | --- | --- | --- |
|  | (n=3,417) | | | (n=3,354) | | |
|  | **Pregnancies** | **Cycles** | **Adjusted FR**  **(95% CI)** | **Pregnancies** | **Cycles** | **Adjusted FR**  **(95% CI)** |
| **ICE_income_** |  |  |  |  |  |  |
| Q1 (most disadvantaged) | 294 | 2,328 | 0.84 (0.74-0.97) | 290 | 2,236 | 0.86 (0.75-0.98) |
| Q2 | 413 | 2,963 | 0.88 (0.78-0.99) | 410 | 2,914 | 0.88 (0.78-0.99) |
| Q3 | 481 | 3,230 | 0.94 (0.84-1.05) | 479 | 3,152 | 0.95 (0.85-1.07) |
| Q4 | 529 | 3,369 | 0.96 (0.86-1.07) | 526 | 3,349 | 0.96 (0.86-1.07) |
| Q5 (most privileged) | 555 | 3,344 | 1.00 (Reference) | 555 | 3,326 | 1.00 (Reference) |
| **ICE_white/black_** |  |  |  |  |  |  |
| Q1 (most disadvantaged) | 371 | 2,611 | 0.92 (0.80-1.05) | 370 | 2,542 | 0.92 (0.81-1.06) |
| Q2 | 485 | 3,399 | 0.92 (0.82-1.05) | 482 | 3,335 | 0.92 (0.81-1.04) |
| Q3 | 508 | 3,497 | 0.94 (0.83-1.06) | 507 | 3,455 | 0.93 (0.83-1.06) |
| Q4 | 485 | 3,059 | 1.01 (0.89-1.14) | 480 | 3,036 | 0.99 (0.88-1.12) |
| Q5 (most privileged) | 423 | 2,668 | 1.00 (Reference) | 421 | 2,609 | 1.00 (Reference) |
| **ICE_income + white/black_** |  |  |  |  |  |  |
| Q1 (most disadvantaged) | 343 | 2,407 | 0.91 (0.80-1.04) | 339 | 2,332 | 0.92 (0.81-1.04) |
| Q2 | 391 | 2,977 | 0.82 (0.72-0.92) | 388 | 2,900 | 0.82 (0.73-0.93) |
| Q3 | 428 | 3,056 | 0.87 (0.77-0.97) | 427 | 3,010 | 0.87 (0.77-0.98) |
| Q4 | 553 | 3,531 | 0.94 (0.84-1.04) | 550 | 3,493 | 0.94 (0.84-1.04) |
| Q5 (most privileged) | 557 | 3,263 | 1.00 (Reference) | 556 | 3,242 | 1.00 (Reference) |
| **ICE_education_** |  |  |  |  |  |  |
| Q1 (most disadvantaged) | 239 | 1,799 | 0.87 (0.76-1.00) | 237 | 1,721 | 0.89 (0.78-1.03) |
| Q2 | 353 | 2,690 | 0.82 (0.72-0.93) | 348 | 2,592 | 0.83 (0.74-0.94) |
| Q3 | 481 | 3,243 | 0.92 (0.82-1.02) | 480 | 3,209 | 0.92 (0.83-1.03) |
| Q4 | 534 | 3,351 | 0.99 (0.89-1.10) | 532 | 3,339 | 0.99 (0.89-1.10) |
| Q5 (most privileged) | 665 | 4,151 | 1.00 (Reference) | 663 | 4,116 | 1.00 (Reference) |
| **ICE_white/hispanic_** |  |  |  |  |  |  |
| Q1 (most disadvantaged) | 371 | 2,607 | 0.91 (0.80-1.04) | 370 | 2,535 | 0.91 (0.80-1.05) |
| Q2 | 487 | 3,185 | 0.98 (0.87-1.11) | 486 | 3,131 | 0.98 (0.87-1.11) |
| Q3 | 515 | 3,669 | 0.92 (0.82-1.04) | 510 | 3,624 | 0.91 (0.80-1.03) |
| Q4 | 469 | 3,061 | 0.98 (0.87-1.11) | 466 | 3,036 | 0.97 (0.86-1.10) |
| Q5 (most privileged) | 430 | 2,712 | 1.00 (Reference) | 428 | 2,651 | 1.00 (Reference) |
| **ICE_white/asian_** |  |  |  |  |  |  |
| Q1 (most disadvantaged) | 414 | 2,986 | 0.91 (0.80-1.04) | 412 | 2,911 | 0.91 (0.80-1.04) |
| Q2 | 477 | 3,110 | 1.02 (0.90-1.16) | 476 | 3,044 | 1.03 (0.91-1.16) |
| Q3 | 514 | 3,556 | 0.96 (0.85-1.09) | 512 | 3,522 | 0.95 (0.84-1.08) |
| Q4 | 454 | 2,940 | 1.02 (0.90-1.15) | 450 | 2,907 | 1.01 (0.89-1.14) |
| Q5 (most privileged) | 413 | 2,642 | 1.00 (Reference) | 410 | 2,593 | 1.00 (Reference) |
| **ICE_income + white/hispanic_** |  |  |  |  |  |  |
| Q1 (most disadvantaged) | 317 | 2,462 | 0.83 (0.73-0.95) | 313 | 2,370 | 0.84 (0.74-0.96) |
| Q2 | 380 | 2,769 | 0.84 (0.74-0.95) | 377 | 2,728 | 0.84 (0.74-0.95) |
| Q3 | 496 | 3,363 | 0.90 (0.81-1.01) | 494 | 3,275 | 0.91 (0.82-1.02) |
| Q4 | 515 | 3,357 | 0.91 (0.82-1.02) | 512 | 3,341 | 0.91 (0.81-1.01) |
| Q5 (most privileged) | 564 | 3,283 | 1.00 (Reference) | 564 | 3,263 | 1.00 (Reference) |
| **ICE_income + white/asian_** |  |  |  |  |  |  |
| Q1 (most disadvantaged) | 347 | 2,484 | 0.90 (0.79-1.02) | 341 | 2,402 | 0.90 (0.80-1.03) |
| Q2 | 382 | 2,965 | 0.81 (0.72-0.92) | 381 | 2,870 | 0.83 (0.73-0.94) |
| Q3 | 438 | 3,054 | 0.89 (0.79-1.00) | 438 | 3,036 | 0.89 (0.79-1.00) |
| Q4 | 552 | 3,458 | 0.97 (0.87-1.08) | 548 | 3,417 | 0.97 (0.87-1.08) |
| Q5 (most privileged) | 553 | 3,273 | 1.00 (Reference) | 552 | 3,252 | 1.00 (Reference) |

Note: CI = confidence interval; FR = fecundability ratio; PRESTO = Pregnancy Study Online; Adjusted for age, calendar year of enrollment, and geographic region of residence

**eTable 12.** Associations between economic, racial, and racialized economic segregation with fecundability stratified by body mass index, PRESTO 2013-2022

|  | | **Body Mass Index** | | | | | | | | | |
| --- | --- | --- | --- | --- | --- | --- | --- | --- | --- | --- | --- |
|  | | **<25 kg/m^2^** | | | **25-29 kg/m^2^** | | | **≥30 kg/m^2^** | | | |
|  | | (n=4,655) | | | (n=2,505) | | | (n=3,278) | | | |
|  | **Pregnancies** | | **Cycles** | **Adjusted FR**  **(95% CI)** | **Pregnancies** | **Cycles** | **Adjusted FR**  **(95% CI)** | **Pregnancies** | **Cycles** | **Adjusted FR**  **(95% CI)** |  |
| **ICE_income_** |  | |  |  |  |  |  |  |  |  |  |
| Q1 (most disadvantaged) | 375 | | 2,573 | 0.89 (0.79-0.99) | 234 | 1,603 | 0.92 (0.78-1.08) | 303 | 3,440 | 0.75 (0.64-0.89) |  |
| Q2 | 533 | | 3,254 | 0.93 (0.84-1.03) | 281 | 1,906 | 0.88 (0.76-1.02) | 316 | 3,079 | 0.83 (0.71-0.98) |  |
| Q3 | 636 | | 3,801 | 0.95 (0.86-1.04) | 335 | 2,216 | 0.88 (0.77-1.02) | 318 | 2,898 | 0.87 (0.74-1.02) |  |
| Q4 | 748 | | 4,097 | 1.00 (0.92-1.09) | 354 | 2,249 | 0.93 (0.82-1.07) | 299 | 2,588 | 0.88 (0.75-1.03) |  |
| Q5 (most privileged) | 883 | | 4,778 | 1.00 (Reference) | 371 | 2,154 | 1.00 (Reference) | 252 | 1,939 | 1.00 (Reference) |  |
| **ICE_white/black_** |  | |  |  |  |  |  |  |  |  |  |
| Q1 (most disadvantaged) | 520 | | 3,155 | 0.94 (0.84-1.05) | 243 | 1,839 | 0.88 (0.74-1.04) | 280 | 2,869 | 0.91 (0.77-1.07) |  |
| Q2 | 645 | | 3,909 | 0.91 (0.82-1.01) | 342 | 2,243 | 0.96 (0.82-1.11) | 301 | 2,706 | 1.02 (0.87-1.19) |  |
| Q3 | 709 | | 4,272 | 0.91 (0.82-1.01) | 337 | 2,102 | 1.01 (0.87-1.17) | 282 | 2,628 | 1.01 (0.86-1.18) |  |
| Q4 | 709 | | 3,910 | 0.98 (0.89-1.08) | 343 | 2,092 | 1.01 (0.88-1.17) | 285 | 2,628 | 1.01 (0.86-1.17) |  |
| Q5 (most privileged) | 592 | | 3,257 | 1.00 (Reference) | 310 | 1,852 | 1.00 (Reference) | 340 | 3,113 | 1.00 (Reference) |  |
| **ICE_income + white/black_** |  | |  |  |  |  |  |  |  |  |  |
| Q1 (most disadvantaged) | 406 | | 2,719 | 0.88 (0.79-0.98) | 236 | 1,733 | 0.83 (0.71-0.98) | 295 | 3,216 | 0.77 (0.65-0.91) |  |
| Q2 | 523 | | 3,298 | 0.88 (0.80-0.98) | 292 | 1,867 | 0.90 (0.77-1.04) | 334 | 3,296 | 0.84 (0.71-0.98) |  |
| Q3 | 606 | | 3,683 | 0.91 (0.83-1.00) | 299 | 2,035 | 0.84 (0.72-0.97) | 299 | 2,915 | 0.81 (0.69-0.95) |  |
| Q4 | 733 | | 4,090 | 0.96 (0.88-1.05) | 391 | 2,494 | 0.89 (0.78-1.01) | 296 | 2,474 | 0.93 (0.79-1.10) |  |
| Q5 (most privileged) | 907 | | 4,713 | 1.00 (Reference) | 357 | 1,999 | 1.00 (Reference) | 264 | 2,043 | 1.00 (Reference) |  |
| **ICE_education_** |  | |  |  |  |  |  |  |  |  |  |
| Q1 (most disadvantaged) | 325 | | 2,158 | 0.93 (0.82-1.04) | 216 | 1,476 | 0.88 (0.75-1.04) | 314 | 3,440 | 0.69 (0.59-0.81) |  |
| Q2 | 455 | | 2,928 | 0.91 (0.82-1.01) | 278 | 1,923 | 0.81 (0.70-0.94) | 335 | 3,352 | 0.74 (0.63-0.87) |  |
| Q3 | 619 | | 3,624 | 0.99 (0.91-1.09) | 360 | 2,441 | 0.84 (0.73-0.96) | 314 | 2,893 | 0.78 (0.66-0.92) |  |
| Q4 | 771 | | 4,221 | 1.04 (0.96-1.14) | 359 | 2,215 | 0.92 (0.81-1.06) | 293 | 2,588 | 0.81 (0.69-0.95) |  |
| Q5 (most privileged) | 1,005 | | 5,572 | 1.00 (Reference) | 362 | 2,073 | 1.00 (Reference) | 232 | 1,671 | 1.00 (Reference) |  |
| **ICE_white/hispanic_** |  | |  |  |  |  |  |  |  |  |  |
| Q1 (most disadvantaged) | 521 | | 3,258 | 0.93 (0.83-1.04) | 261 | 2,019 | 0.84 (0.71-0.99) | 285 | 2,737 | 0.98 (0.83-1.15) |  |
| Q2 | 679 | | 4,017 | 0.96 (0.86-1.07) | 324 | 2,021 | 1.01 (0.87-1.17) | 284 | 2,711 | 0.95 (0.81-1.12) |  |
| Q3 | 717 | | 4,196 | 0.95 (0.85-1.05) | 320 | 2,070 | 0.99 (0.85-1.15) | 290 | 2,638 | 1.04 (0.89-1.21) |  |
| Q4 | 681 | | 3,808 | 0.99 (0.89-1.09) | 346 | 2,065 | 1.02 (0.88-1.17) | 284 | 2,666 | 0.98 (0.84-1.15) |  |
| Q5 (most privileged) | 577 | | 3,224 | 1.00 (Reference) | 324 | 1,953 | 1.00 (Reference) | 345 | 3,192 | 1.00 (Reference) |  |
| **ICE_white/asian_** |  | |  |  |  |  |  |  |  |  |  |
| Q1 (most disadvantaged) | 602 | | 3,588 | 0.99 (0.89-1.11) | 265 | 2,012 | 0.86 (0.73-1.00) | 269 | 2,766 | 0.92 (0.78-1.08) |  |
| Q2 | 653 | | 3,869 | 0.99 (0.89-1.10) | 325 | 2,128 | 0.96 (0.83-1.12) | 285 | 2,567 | 1.02 (0.87-1.20) |  |
| Q3 | 686 | | 4,215 | 0.93 (0.84-1.04) | 321 | 2,027 | 0.99 (0.86-1.15) | 307 | 2,661 | 1.11 (0.95-1.29) |  |
| Q4 | 683 | | 3,660 | 1.06 (0.96-1.18) | 351 | 2,090 | 1.02 (0.89-1.18) | 285 | 2,794 | 0.97 (0.83-1.13) |  |
| Q5 (most privileged) | 551 | | 3,171 | 1.00 (Reference) | 313 | 1,871 | 1.00 (Reference) | 342 | 3,156 | 1.00 (Reference) |  |
| **ICE_income + white/hispanic_** |  | |  |  |  |  |  |  |  |  |  |
| Q1 (most disadvantaged) | 389 | | 2,657 | 0.87 (0.78-0.97) | 239 | 1,630 | 0.87 (0.75-1.02) | 295 | 3,365 | 0.76 (0.64-0.89) |  |
| Q2 | 511 | | 3,163 | 0.91 (0.82-1.00) | 271 | 1,923 | 0.80 (0.69-0.93) | 323 | 3,040 | 0.88 (0.75-1.03) |  |
| Q3 | 646 | | 3,970 | 0.91 (0.83-1.00) | 327 | 2,110 | 0.86 (0.75-0.99) | 318 | 2,918 | 0.89 (0.76-1.04) |  |
| Q4 | 747 | | 4,069 | 0.98 (0.90-1.07) | 356 | 2,389 | 0.84 (0.73-0.96) | 290 | 2,564 | 0.88 (0.75-1.03) |  |
| Q5 (most privileged) | 882 | | 4,644 | 1.00 (Reference) | 382 | 2,076 | 1.00 (Reference) | 262 | 2,057 | 1.00 (Reference) |  |
| **ICE_income + white/asian_** |  | |  |  |  |  |  |  |  |  |  |
| Q1 (most disadvantaged) | 420 | | 2,781 | 0.90 (0.81-1.00) | 239 | 1,705 | 0.84 (0.72-0.98) | 299 | 3,335 | 0.77 (0.65-0.91) |  |
| Q2 | 498 | | 3,183 | 0.88 (0.80-0.98) | 292 | 1,982 | 0.86 (0.74-0.99) | 330 | 3,239 | 0.84 (0.72-0.99) |  |
| Q3 | 607 | | 3,810 | 0.89 (0.81-0.98) | 299 | 1,940 | 0.87 (0.76-1.01) | 305 | 2,831 | 0.87 (0.74-1.02) |  |
| Q4 | 744 | | 3,965 | 1.01 (0.93-1.11) | 386 | 2,485 | 0.88 (0.77-1.01) | 298 | 2,526 | 0.93 (0.80-1.10) |  |
| Q5 (most privileged) | 906 | | 4,764 | 1.00 (Reference) | 359 | 2,016 | 1.00 (Reference) | 256 | 2,013 | 1.00 (Reference) |  |

Note: CI = confidence interval; FR = fecundability ratio; PRESTO = Pregnancy Study Online; Adjusted for age, calendar year of enrollment, and geographic region of residence

**eTable 13.** Associations between economic, racial, and racialized economic segregation with fecundability stratified by participants’ race/ethnicity, PRESTO 2013-2022

|  | | **Race/Ethnicity** | | | | | | | | | |
| --- | --- | --- | --- | --- | --- | --- | --- | --- | --- | --- | --- |
|  | | **Non-Hispanic White** | | | **Non-Hispanic Other Race^a^** | | | **Hispanic** | | | |
|  | | (n=8,707) | | | (n=959) | | | (n=772) | | | |
|  | **Pregnancies** | | **Cycles** | **Adjusted FR**  **(95% CI)** | **Pregnancies** | **Cycles** | **Adjusted FR**  **(95% CI)** | **Pregnancies** | **Cycles** | **Adjusted FR**  **(95% CI)** |  |
| **ICE_income_** |  | |  |  |  |  |  |  |  |  |  |
| Q1 (most disadvantaged) | 753 | | 5,985 | 0.82 (0.75-0.89) | 83 | 972 | 0.78 (0.59-1.04) | 76 | 659 | 0.68 (0.50-0.92) |  |
| Q2 | 977 | | 6,951 | 0.86 (0.79-0.93) | 81 | 725 | 0.93 (0.70-1.23) | 72 | 563 | 0.76 (0.57-1.02) |  |
| Q3 | 1,106 | | 7,480 | 0.89 (0.83-0.96) | 94 | 857 | 0.91 (0.69-1.19) | 89 | 578 | 0.93 (0.71-1.22) |  |
| Q4 | 1,228 | | 7,583 | 0.95 (0.89-1.02) | 82 | 697 | 0.90 (0.69-1.19) | 91 | 654 | 0.78 (0.60-1.03) |  |
| Q5 (most privileged) | 1,296 | | 7,470 | 1.00 (Reference) | 108 | 793 | 1.00 (Reference) | 102 | 608 | 1.00 (Reference) |  |
| **ICE_white/black_** |  | |  |  |  |  |  |  |  |  |  |
| Q1 (most disadvantaged) | 777 | | 5,416 | 0.98 (0.90-1.07) | 141 | 1,490 | 0.84 (0.60-1.18) | 125 | 957 | 0.72 (0.52-0.99) |  |
| Q2 | 1,088 | | 7,206 | 1.00 (0.92-1.09) | 96 | 833 | 0.91 (0.64-1.30) | 104 | 819 | 0.69 (0.50-0.96) |  |
| Q3 | 1,137 | | 7,581 | 1.00 (0.93-1.08) | 95 | 783 | 0.95 (0.67-1.36) | 96 | 638 | 0.82 (0.59-1.13) |  |
| Q4 | 1,208 | | 7,657 | 1.04 (0.96-1.12) | 73 | 574 | 0.95 (0.65-1.37) | 56 | 399 | 0.75 (0.52-1.07) |  |
| Q5 (most privileged) | 1,150 | | 7,609 | 1.00 (Reference) | 43 | 364 | 1.00 (Reference) | 49 | 249 | 1.00 (Reference) |  |
| **ICE_income + white/black_** |  | |  |  |  |  |  |  |  |  |  |
| Q1 (most disadvantaged) | 720 | | 5,583 | 0.82 (0.75-0.90) | 119 | 1,262 | 0.83 (0.63-1.10) | 98 | 823 | 0.64 (0.48-0.84) |  |
| Q2 | 993 | | 7,128 | 0.85 (0.79-0.92) | 69 | 759 | 0.75 (0.55-1.03) | 87 | 574 | 0.79 (0.59-1.04) |  |
| Q3 | 1,055 | | 7,364 | 0.85 (0.79-0.91) | 82 | 725 | 0.90 (0.67-1.21) | 67 | 544 | 0.71 (0.53-0.95) |  |
| Q4 | 1,237 | | 7,698 | 0.94 (0.88-1.01) | 100 | 723 | 1.00 (0.75-1.33) | 83 | 637 | 0.67 (0.51-0.89) |  |
| Q5 (most privileged) | 1,355 | | 7,696 | 1.00 (Reference) | 78 | 575 | 1.00 (Reference) | 95 | 484 | 1.00 (Reference) |  |
| **ICE_education_** |  | |  |  |  |  |  |  |  |  |  |
| Q1 (most disadvantaged) | 718 | | 5,543 | 0.82 (0.75-0.89) | 65 | 794 | 0.72 (0.54-0.97) | 72 | 737 | 0.53 (0.39-0.72) |  |
| Q2 | 913 | | 6,872 | 0.80 (0.74-0.86) | 76 | 758 | 0.87 (0.66-1.15) | 79 | 573 | 0.79 (0.60-1.05) |  |
| Q3 | 1,130 | | 7,510 | 0.89 (0.83-0.96) | 77 | 784 | 0.81 (0.61-1.08) | 86 | 664 | 0.77 (0.58-1.01) |  |
| Q4 | 1,220 | | 7,621 | 0.94 (0.88-1.01) | 104 | 820 | 1.00 (0.78-1.29) | 99 | 583 | 0.94 (0.73-1.22) |  |
| Q5 (most privileged) | 1,379 | | 7,923 | 1.00 (Reference) | 126 | 888 | 1.00 (Reference) | 94 | 505 | 1.00 (Reference) |  |
| **ICE_white/hispanic_** |  | |  |  |  |  |  |  |  |  |  |
| Q1 (most disadvantaged) | 787 | | 5,502 | 0.98 (0.90-1.08) | 131 | 1,361 | 0.80 (0.57-1.11) | 149 | 1,151 | 0.73 (0.53-1.02) |  |
| Q2 | 1,069 | | 6,981 | 1.04 (0.96-1.13) | 116 | 977 | 0.91 (0.66-1.27) | 102 | 791 | 0.73 (0.52-1.03) |  |
| Q3 | 1,161 | | 7,632 | 1.02 (0.95-1.11) | 88 | 731 | 0.88 (0.63-1.24) | 78 | 541 | 0.84 (0.59-1.19) |  |
| Q4 | 1,192 | | 7,626 | 1.04 (0.96-1.12) | 63 | 577 | 0.83 (0.57-1.19) | 56 | 336 | 0.88 (0.60-1.27) |  |
| Q5 (most privileged) | 1,151 | | 7,728 | 1.00 (Reference) | 50 | 398 | 1.00 (Reference) | 45 | 243 | 1.00 (Reference) |  |
| **ICE_white/asian_** |  | |  |  |  |  |  |  |  |  |  |
| Q1 (most disadvantaged) | 842 | | 5,729 | 1.01 (0.93-1.10) | 149 | 1,540 | 0.94 (0.66-1.35) | 145 | 1,097 | 0.76 (0.55-1.05) |  |
| Q2 | 1,055 | | 6,926 | 1.05 (0.97-1.14) | 110 | 881 | 1.15 (0.80-1.66) | 98 | 757 | 0.71 (0.51-1.00) |  |
| Q3 | 1,148 | | 7,620 | 1.03 (0.95-1.11) | 79 | 665 | 1.04 (0.71-1.53) | 87 | 618 | 0.81 (0.58-1.14) |  |
| Q4 | 1,195 | | 7,616 | 1.07 (0.99-1.15) | 70 | 581 | 1.09 (0.74-1.60) | 54 | 347 | 0.82 (0.56-1.20) |  |
| Q5 (most privileged) | 1,120 | | 7,578 | 1.00 (Reference) | 40 | 377 | 1.00 (Reference) | 46 | 243 | 1.00 (Reference) |  |
| **ICE_income + white/hispanic_** |  | |  |  |  |  |  |  |  |  |  |
| Q1 (most disadvantaged) | 743 | | 5,887 | 0.81 (0.74-0.88) | 93 | 1,028 | 0.81 (0.61-1.08) | 87 | 737 | 0.63 (0.47-0.84) |  |
| Q2 | 932 | | 6,676 | 0.84 (0.78-0.91) | 88 | 846 | 0.92 (0.68-1.23) | 85 | 604 | 0.75 (0.56-0.99) |  |
| Q3 | 1,118 | | 7,564 | 0.88 (0.82-0.95) | 92 | 864 | 0.87 (0.66-1.15) | 81 | 570 | 0.79 (0.59-1.04) |  |
| Q4 | 1,220 | | 7,681 | 0.92 (0.86-0.99) | 90 | 667 | 1.04 (0.79-1.38) | 83 | 674 | 0.67 (0.51-0.88) |  |
| Q5 (most privileged) | 1,347 | | 7,661 | 1.00 (Reference) | 85 | 639 | 1.00 (Reference) | 94 | 477 | 1.00 (Reference) |  |
| **ICE_income + white/asian_** |  | |  |  |  |  |  |  |  |  |  |
| Q1 (most disadvantaged) | 741 | | 5,784 | 0.82 (0.75-0.89) | 101 | 1,159 | 0.77 (0.58-1.03) | 116 | 878 | 0.70 (0.54-0.93) |  |
| Q2 | 970 | | 7,020 | 0.85 (0.79-0.92) | 84 | 847 | 0.81 (0.60-1.09) | 66 | 537 | 0.67 (0.49-0.91) |  |
| Q3 | 1,056 | | 7,334 | 0.86 (0.80-0.93) | 83 | 694 | 0.93 (0.69-1.24) | 72 | 553 | 0.71 (0.53-0.95) |  |
| Q4 | 1,243 | | 7,583 | 0.97 (0.90-1.04) | 99 | 752 | 0.95 (0.71-1.25) | 86 | 641 | 0.71 (0.54-0.93) |  |
| Q5 (most privileged) | 1,350 | | 7,748 | 1.00 (Reference) | 81 | 592 | 1.00 (Reference) | 90 | 453 | 1.00 (Reference) |  |

Note: CI = confidence interval; FR = fecundability ratio; PRESTO = Pregnancy Study Online; Adjusted for age, calendar year of enrollment, and geographic region of residence; ^a^Includes Black, Asian/Native Hawaiian/Pacific Islander, Multiracial, American Indian, Alaskan Native, Indigenous, Middle Eastern or North African, some other race, or missing race

**eTable 14.** Associations between economic, racial, and racialized economic segregation with fecundability stratified by educational attainment, PRESTO 2013-2022

|  | **Educational Attainment** | | | | | | | | | | | |
| --- | --- | --- | --- | --- | --- | --- | --- | --- | --- | --- | --- | --- |
|  | **≤12 Years** | | | **13-15 Years** | | | **16 Years** | | | **≥17 Years** | | |
|  | (n=510) | | | (n=2,063) | | | (n=3,383) | | | (n=4,482) | | |
|  | **Pregnancies** | **Cycles** | **Adjusted FR**  **(95% CI)** | **Pregnancies** | **Cycles** | **Adjusted FR**  **(95% CI)** | **Pregnancies** | **Cycles** | **Adjusted FR**  **(95% CI)** | **Pregnancies** | **Cycles** | **Adjusted FR**  **(95% CI)** |
| **ICE_income_** |  |  |  |  |  |  |  |  |  |  |  |  |
| Q1 (most disadvantaged) | 51 | 693 | 0.65 (0.38-1.14) | 230 | 2,344 | 0.90 (0.72-1.14) | 278 | 2,142 | 0.88 (0.76-1.02) | 353 | 2,437 | 0.85 (0.75-0.95) |
| Q2 | 38 | 442 | 0.79 (0.44-1.40) | 230 | 2,142 | 0.91 (0.73-1.15) | 381 | 2,796 | 0.88 (0.77-1.00) | 481 | 2,859 | 0.92 (0.83-1.02) |
| Q3 | 43 | 295 | 1.18 (0.68-2.05) | 184 | 1,738 | 0.92 (0.72-1.16) | 447 | 3,227 | 0.89 (0.78-1.00) | 615 | 3,655 | 0.92 (0.84-1.02) |
| Q4 | 25 | 194 | 0.97 (0.53-1.79) | 172 | 1,255 | 1.12 (0.88-1.42) | 486 | 3,103 | 0.97 (0.86-1.09) | 718 | 4,382 | 0.90 (0.83-0.99) |
| Q5 (most privileged) | 19 | 152 | 1.00 (Reference) | 112 | 955 | 1.00 (Reference) | 473 | 2,940 | 1.00 (Reference) | 902 | 4,824 | 1.00 (Reference) |
| **ICE_white/black_** |  |  |  |  |  |  |  |  |  |  |  |  |
| Q1 (most disadvantaged) | 33 | 381 | 1.08 (0.66-1.75) | 194 | 1,831 | 0.94 (0.77-1.14) | 315 | 2,258 | 0.97 (0.84-1.12) | 501 | 3,393 | 0.85 (0.76-0.95) |
| Q2 | 27 | 295 | 0.90 (0.54-1.50) | 153 | 1,463 | 0.90 (0.73-1.10) | 409 | 2,976 | 0.94 (0.83-1.07) | 699 | 4,124 | 0.93 (0.84-1.03) |
| Q3 | 34 | 324 | 1.18 (0.74-1.87) | 177 | 1,587 | 0.99 (0.81-1.20) | 434 | 3,184 | 0.95 (0.84-1.08) | 683 | 3,907 | 0.94 (0.84-1.04) |
| Q4 | 38 | 314 | 1.47 (0.94-2.31) | 176 | 1,527 | 1.00 (0.83-1.22) | 462 | 2,898 | 1.07 (0.95-1.21) | 661 | 3,891 | 0.92 (0.83-1.02) |
| Q5 (most privileged) | 44 | 462 | 1.00 (Reference) | 228 | 2,026 | 1.00 (Reference) | 445 | 2,892 | 1.00 (Reference) | 525 | 2,842 | 1.00 (Reference) |
| **ICE_income + white/black_** |  |  |  |  |  |  |  |  |  |  |  |  |
| Q1 (most disadvantaged) | 41 | 515 | 0.88 (0.48-1.59) | 228 | 2,266 | 0.77 (0.62-0.95) | 277 | 2,218 | 0.87 (0.75-1.00) | 391 | 2,669 | 0.83 (0.74-0.92) |
| Q2 | 53 | 599 | 0.97 (0.55-1.72) | 213 | 2,017 | 0.78 (0.62-0.97) | 387 | 2,847 | 0.90 (0.79-1.02) | 496 | 2,998 | 0.89 (0.80-0.98) |
| Q3 | 30 | 273 | 1.15 (0.63-2.12) | 192 | 1,873 | 0.74 (0.60-0.93) | 434 | 3,144 | 0.90 (0.79-1.01) | 548 | 3,343 | 0.87 (0.79-0.96) |
| Q4 | 34 | 213 | 1.59 (0.89-2.84) | 172 | 1,407 | 0.85 (0.68-1.07) | 490 | 2,965 | 1.05 (0.93-1.18) | 724 | 4,473 | 0.86 (0.79-0.94) |
| Q5 (most privileged) | 18 | 176 | 1.00 (Reference) | 123 | 871 | 1.00 (Reference) | 477 | 3,034 | 1.00 (Reference) | 910 | 4,674 | 1.00 (Reference) |
| **ICE_education_** |  |  |  |  |  |  |  |  |  |  |  |  |
| Q1 (most disadvantaged) | 58 | 696 | 0.63 (0.37-1.06) | 245 | 2,271 | 0.82 (0.65-1.04) | 259 | 2,061 | 0.86 (0.74-0.99) | 293 | 2,046 | 0.85 (0.76-0.96) |
| Q2 | 46 | 468 | 0.66 (0.39-1.12) | 233 | 2,456 | 0.75 (0.59-0.95) | 391 | 2,809 | 0.87 (0.76-0.99) | 398 | 2,470 | 0.90 (0.81-1.00) |
| Q3 | 34 | 306 | 0.69 (0.38-1.23) | 223 | 1,902 | 0.86 (0.68-1.09) | 456 | 3,327 | 0.89 (0.79-1.00) | 580 | 3,423 | 0.95 (0.86-1.04) |
| Q4 | 21 | 188 | 0.79 (0.43-1.47) | 138 | 1,165 | 0.90 (0.70-1.17) | 513 | 3,179 | 1.03 (0.92-1.16) | 751 | 4,492 | 0.93 (0.86-1.02) |
| Q5 (most privileged) | 17 | 118 | 1.00 (Reference) | 89 | 640 | 1.00 (Reference) | 446 | 2,832 | 1.00 (Reference) | 1047 | 5,726 | 1.00 (Reference) |
| **ICE_white/hispanic_** |  |  |  |  |  |  |  |  |  |  |  |  |
| Q1 (most disadvantaged) | 28 | 337 | 0.99 (0.61-1.61) | 187 | 1,724 | 0.95 (0.77-1.16) | 332 | 2,540 | 0.95 (0.82-1.09) | 520 | 3,413 | 0.88 (0.78-0.98) |
| Q2 | 31 | 305 | 1.04 (0.63-1.71) | 168 | 1,661 | 0.86 (0.70-1.05) | 411 | 2,835 | 1.03 (0.90-1.18) | 677 | 3,948 | 0.96 (0.86-1.07) |
| Q3 | 31 | 324 | 0.89 (0.55-1.46) | 165 | 1,439 | 1.04 (0.85-1.26) | 435 | 3,035 | 1.04 (0.91-1.18) | 696 | 4,106 | 0.93 (0.84-1.03) |
| Q4 | 42 | 361 | 1.28 (0.83-1.98) | 183 | 1,617 | 0.96 (0.79-1.16) | 450 | 2,838 | 1.11 (0.98-1.25) | 636 | 3,723 | 0.93 (0.84-1.03) |
| Q5 (most privileged) | 44 | 449 | 1.00 (Reference) | 225 | 1,993 | 1.00 (Reference) | 437 | 2,960 | 1.00 (Reference) | 540 | 2,967 | 1.00 (Reference) |
| **ICE_white/asian_** |  |  |  |  |  |  |  |  |  |  |  |  |
| Q1 (most disadvantaged) | 26 | 318 | 0.97 (0.59-1.59) | 177 | 1,641 | 0.96 (0.79-1.18) | 346 | 2,540 | 0.99 (0.86-1.14) | 587 | 3,867 | 0.89 (0.79-0.99) |
| Q2 | 26 | 254 | 1.10 (0.67-1.83) | 155 | 1,550 | 0.85 (0.69-1.05) | 387 | 2,802 | 0.99 (0.87-1.13) | 695 | 3,958 | 1.01 (0.91-1.12) |
| Q3 | 36 | 390 | 0.89 (0.56-1.41) | 179 | 1,503 | 1.09 (0.90-1.32) | 458 | 3,114 | 1.06 (0.93-1.20) | 641 | 3,896 | 0.92 (0.83-1.03) |
| Q4 | 40 | 322 | 1.40 (0.90-2.18) | 197 | 1,764 | 0.98 (0.81-1.18) | 440 | 2,835 | 1.11 (0.98-1.25) | 642 | 3,623 | 0.99 (0.89-1.10) |
| Q5 (most privileged) | 48 | 492 | 1.00 (Reference) | 220 | 1,976 | 1.00 (Reference) | 434 | 2,917 | 1.00 (Reference) | 504 | 2,813 | 1.00 (Reference) |
| **ICE_income + white/hispanic_** |  |  |  |  |  |  |  |  |  |  |  |  |
| Q1 (most disadvantaged) | 47 | 640 | 0.82 (0.47-1.44) | 229 | 2,278 | 0.78 (0.63-0.97) | 286 | 2,200 | 0.89 (0.77-1.02) | 361 | 2,534 | 0.81 (0.73-0.91) |
| Q2 | 42 | 469 | 0.97 (0.55-1.70) | 224 | 2,042 | 0.79 (0.64-0.98) | 367 | 2,707 | 0.88 (0.77-1.00) | 472 | 2,908 | 0.88 (0.79-0.98) |
| Q3 | 33 | 263 | 1.34 (0.76-2.38) | 183 | 1,809 | 0.75 (0.60-0.94) | 432 | 3,200 | 0.87 (0.77-0.98) | 643 | 3,726 | 0.93 (0.84-1.02) |
| Q4 | 35 | 213 | 1.54 (0.87-2.71) | 166 | 1,396 | 0.84 (0.67-1.05) | 480 | 2,995 | 1.00 (0.89-1.13) | 712 | 4,418 | 0.86 (0.79-0.94) |
| Q5 (most privileged) | 19 | 191 | 1.00 (Reference) | 126 | 909 | 1.00 (Reference) | 500 | 3,106 | 1.00 (Reference) | 881 | 4,571 | 1.00 (Reference) |
| **ICE_income + white/asian_** |  |  |  |  |  |  |  |  |  |  |  |  |
| Q1 (most disadvantaged) | 42 | 555 | 0.87 (0.48-1.56) | 249 | 2,422 | 0.78 (0.63-0.97) | 283 | 2,229 | 0.87 (0.75-1.00) | 384 | 2,615 | 0.84 (0.76-0.94) |
| Q2 | 43 | 514 | 0.92 (0.51-1.65) | 201 | 1,993 | 0.74 (0.59-0.93) | 387 | 2,929 | 0.88 (0.77-1.00) | 489 | 2,968 | 0.90 (0.81-1.00) |
| Q3 | 38 | 311 | 1.29 (0.72-2.30) | 182 | 1,722 | 0.77 (0.62-0.96) | 420 | 3,046 | 0.89 (0.78-1.01) | 571 | 3,502 | 0.88 (0.80-0.97) |
| Q4 | 36 | 220 | 1.63 (0.91-2.91) | 173 | 1,423 | 0.86 (0.69-1.08) | 501 | 3,002 | 1.05 (0.94-1.18) | 718 | 4,331 | 0.90 (0.82-0.98) |
| Q5 (most privileged) | 17 | 176 | 1.00 (Reference) | 123 | 874 | 1.00 (Reference) | 474 | 3,002 | 1.00 (Reference) | 907 | 4,741 | 1.00 (Reference) |

Note: CI = confidence interval; FR = fecundability ratio; PRESTO = Pregnancy Study Online; Adjusted for age, calendar year of enrollment, and geographic region of residence

**eTable 15.** Associations between economic, racial, and racialized economic segregation with fecundability stratified by household income, PRESTO 2013-2022

|  | **Household Income** | | | | | | | | | | | |
| --- | --- | --- | --- | --- | --- | --- | --- | --- | --- | --- | --- | --- |
|  | **<$50,000** | | | **$50,000-$99,999** | | | **$100,000-$149,999** | | | **≥$150,000** | | |
|  | (n=1,913) | | | (n=3,682) | | | (n=2,660) | | | (n=2,183) | | |
|  | **Pregnancies** | **Cycles** | **Adjusted FR**  **(95% CI)** | **Pregnancies** | **Cycles** | **Adjusted FR**  **(95% CI)** | **Pregnancies** | **Cycles** | **Adjusted FR**  **(95% CI)** | **Pregnancies** | **Cycles** | **Adjusted FR**  **(95% CI)** |
| **ICE_income_** |  |  |  |  |  |  |  |  |  |  |  |  |
| Q1 (most disadvantaged) | 271 | 2,712 | 0.72 (0.56-0.93) | 395 | 3,274 | 0.85 (0.74-0.98) | 165 | 1,102 | 0.97 (0.82-1.14) | 81 | 528 | 0.95 (0.77-1.18) |
| Q2 | 225 | 2,043 | 0.75 (0.58-0.97) | 472 | 3,562 | 0.89 (0.77-1.02) | 298 | 1,804 | 0.97 (0.85-1.11) | 135 | 830 | 0.94 (0.80-1.12) |
| Q3 | 161 | 1,330 | 0.80 (0.62-1.05) | 520 | 3,736 | 0.91 (0.80-1.05) | 363 | 2,265 | 0.97 (0.85-1.10) | 245 | 1,584 | 0.90 (0.79-1.03) |
| Q4 | 104 | 894 | 0.77 (0.57-1.04) | 449 | 3,019 | 0.97 (0.85-1.11) | 440 | 2,658 | 0.97 (0.86-1.09) | 408 | 2,363 | 0.98 (0.88-1.10) |
| Q5 (most privileged) | 73 | 490 | 1.00 (Reference) | 303 | 1,937 | 1.00 (Reference) | 481 | 2,801 | 1.00 (Reference) | 649 | 3,643 | 1.00 (Reference) |
| **ICE_white/black_** |  |  |  |  |  |  |  |  |  |  |  |  |
| Q1 (most disadvantaged) | 179 | 1,760 | 0.91 (0.73-1.13) | 354 | 2,935 | 0.89 (0.78-1.02) | 258 | 1,639 | 0.98 (0.83-1.15) | 252 | 1,529 | 0.95 (0.80-1.14) |
| Q2 | 143 | 1,157 | 0.96 (0.77-1.20) | 428 | 3,258 | 0.94 (0.83-1.07) | 356 | 2,273 | 0.93 (0.81-1.07) | 361 | 2,170 | 0.95 (0.81-1.12) |
| Q3 | 161 | 1,590 | 0.88 (0.71-1.09) | 416 | 2,867 | 1.01 (0.89-1.14) | 382 | 2,341 | 0.95 (0.83-1.09) | 369 | 2,204 | 0.97 (0.83-1.14) |
| Q4 | 162 | 1,276 | 1.08 (0.88-1.33) | 450 | 3,083 | 1.04 (0.92-1.17) | 403 | 2,408 | 0.97 (0.85-1.11) | 322 | 1,863 | 0.95 (0.81-1.11) |
| Q5 (most privileged) | 189 | 1,686 | 1.00 (Reference) | 491 | 3,385 | 1.00 (Reference) | 348 | 1,969 | 1.00 (Reference) | 214 | 1,182 | 1.00 (Reference) |
| **ICE_income + white/black_** |  |  |  |  |  |  |  |  |  |  |  |  |
| Q1 (most disadvantaged) | 245 | 2,454 | 0.74 (0.56-0.96) | 380 | 3,170 | 0.79 (0.69-0.91) | 191 | 1,294 | 0.95 (0.81-1.11) | 121 | 750 | 0.96 (0.80-1.15) |
| Q2 | 238 | 2,006 | 0.85 (0.65-1.10) | 474 | 3,683 | 0.82 (0.72-0.93) | 295 | 1,853 | 0.94 (0.82-1.08) | 142 | 919 | 0.91 (0.76-1.07) |
| Q3 | 151 | 1,595 | 0.68 (0.52-0.90) | 460 | 3,386 | 0.83 (0.73-0.95) | 353 | 2,054 | 1.00 (0.88-1.14) | 240 | 1,598 | 0.88 (0.77-1.01) |
| Q4 | 129 | 898 | 0.97 (0.73-1.27) | 470 | 3,192 | 0.90 (0.79-1.03) | 414 | 2,628 | 0.93 (0.83-1.05) | 407 | 2,340 | 0.97 (0.87-1.09) |
| Q5 (most privileged) | 202 | 1,742 | 1.00 (Reference) | 355 | 2,097 | 1.00 (Reference) | 494 | 2,801 | 1.00 (Reference) | 608 | 3,341 | 1.00 (Reference) |
| **ICE_education_** |  |  |  |  |  |  |  |  |  |  |  |  |
| Q1 (most disadvantaged) | 248 | 2,443 | 0.78 (0.61-0.99) | 370 | 3,011 | 0.83 (0.72-0.95) | 155 | 1,080 | 0.89 (0.75-1.06) | 82 | 540 | 0.94 (0.76-1.17) |
| Q2 | 210 | 2,037 | 0.80 (0.63-1.02) | 460 | 3,717 | 0.81 (0.71-0.93) | 272 | 1,623 | 0.94 (0.82-1.08) | 126 | 826 | 0.85 (0.72-1.02) |
| Q3 | 177 | 1,474 | 0.83 (0.64-1.06) | 478 | 3,559 | 0.89 (0.78-1.01) | 411 | 2,570 | 0.93 (0.83-1.06) | 227 | 1,355 | 0.94 (0.82-1.08) |
| Q4 | 114 | 868 | 0.99 (0.76-1.31) | 482 | 3,058 | 1.02 (0.90-1.16) | 460 | 2,813 | 0.95 (0.85-1.07) | 367 | 2,285 | 0.89 (0.79-0.99) |
| Q5 (most privileged) | 85 | 647 | 1.00 (Reference) | 349 | 2,183 | 1.00 (Reference) | 449 | 2,544 | 1.00 (Reference) | 716 | 3,942 | 1.00 (Reference) |
| **ICE_white/hispanic_** |  |  |  |  |  |  |  |  |  |  |  |  |
| Q1 (most disadvantaged) | 177 | 1,635 | 0.89 (0.71-1.10) | 360 | 2,952 | 0.90 (0.78-1.03) | 262 | 1,744 | 0.99 (0.84-1.16) | 268 | 1,683 | 0.94 (0.80-1.12) |
| Q2 | 163 | 1,427 | 0.93 (0.75-1.14) | 406 | 3,098 | 0.94 (0.82-1.07) | 347 | 2,102 | 1.04 (0.90-1.19) | 371 | 2,122 | 1.02 (0.87-1.19) |
| Q3 | 153 | 1,286 | 0.99 (0.80-1.22) | 435 | 2,962 | 1.05 (0.93-1.19) | 390 | 2,527 | 0.95 (0.82-1.08) | 349 | 2,129 | 0.97 (0.83-1.13) |
| Q4 | 142 | 1,394 | 0.85 (0.69-1.05) | 462 | 3,186 | 1.03 (0.91-1.16) | 396 | 2,162 | 1.08 (0.95-1.24) | 311 | 1,797 | 0.97 (0.83-1.14) |
| Q5 (most privileged) | 199 | 1,727 | 1.00 (Reference) | 476 | 3,330 | 1.00 (Reference) | 352 | 2,095 | 1.00 (Reference) | 219 | 1,217 | 1.00 (Reference) |
| **ICE_white/asian_** |  |  |  |  |  |  |  |  |  |  |  |  |
| Q1 (most disadvantaged) | 161 | 1,572 | 0.87 (0.70-1.08) | 349 | 2,917 | 0.88 (0.76-1.00) | 279 | 1,807 | 1.03 (0.88-1.20) | 347 | 2,070 | 1.04 (0.88-1.24) |
| Q2 | 152 | 1,283 | 0.92 (0.74-1.14) | 401 | 2,966 | 0.96 (0.85-1.10) | 355 | 2,201 | 1.03 (0.89-1.19) | 355 | 2,114 | 1.07 (0.91-1.26) |
| Q3 | 164 | 1,495 | 0.93 (0.76-1.15) | 443 | 3,078 | 1.02 (0.91-1.16) | 378 | 2,316 | 1.00 (0.87-1.15) | 329 | 2,014 | 1.01 (0.85-1.19) |
| Q4 | 155 | 1,377 | 0.94 (0.76-1.15) | 460 | 3,216 | 1.01 (0.90-1.14) | 414 | 2,373 | 1.08 (0.94-1.24) | 290 | 1,578 | 1.11 (0.94-1.31) |
| Q5 (most privileged) | 202 | 1,742 | 1.00 (Reference) | 486 | 3,351 | 1.00 (Reference) | 321 | 1,933 | 1.00 (Reference) | 197 | 1,172 | 1.00 (Reference) |
| **ICE_income + white/hispanic_** |  |  |  |  |  |  |  |  |  |  |  |  |
| Q1 (most disadvantaged) | 264 | 2,654 | 0.67 (0.53-0.86) | 389 | 3,200 | 0.81 (0.70-0.92) | 174 | 1,169 | 0.94 (0.80-1.11) | 96 | 629 | 0.96 (0.78-1.17) |
| Q2 | 230 | 1,899 | 0.76 (0.59-0.98) | 440 | 3,468 | 0.81 (0.71-0.92) | 295 | 1,842 | 0.95 (0.83-1.09) | 140 | 917 | 0.90 (0.76-1.07) |
| Q3 | 148 | 1,499 | 0.63 (0.49-0.82) | 502 | 3,555 | 0.89 (0.78-1.01) | 359 | 2,196 | 0.95 (0.84-1.08) | 282 | 1,748 | 0.95 (0.83-1.09) |
| Q4 | 113 | 890 | 0.78 (0.59-1.04) | 448 | 3,160 | 0.88 (0.78-1.01) | 417 | 2,582 | 0.95 (0.84-1.07) | 415 | 2,390 | 0.98 (0.88-1.10) |
| Q5 (most privileged) | 79 | 527 | 1.00 (Reference) | 360 | 2,145 | 1.00 (Reference) | 502 | 2,841 | 1.00 (Reference) | 585 | 3,264 | 1.00 (Reference) |
| **ICE_income + white/asian_** |  |  |  |  |  |  |  |  |  |  |  |  |
| Q1 (most disadvantaged) | 257 | 2,581 | 0.72 (0.56-0.94) | 389 | 3,200 | 0.81 (0.70-0.92) | 191 | 1,221 | 1.00 (0.86-1.17) | 123 | 758 | 0.98 (0.82-1.18) |
| Q2 | 225 | 1,936 | 0.81 (0.62-1.05) | 440 | 3,468 | 0.81 (0.71-0.92) | 300 | 1,926 | 0.94 (0.82-1.08) | 129 | 872 | 0.88 (0.74-1.05) |
| Q3 | 159 | 1,538 | 0.72 (0.54-0.94) | 502 | 3,555 | 0.89 (0.78-1.01) | 344 | 2,082 | 0.98 (0.86-1.12) | 249 | 1,623 | 0.91 (0.79-1.04) |
| Q4 | 123 | 914 | 0.92 (0.69-1.22) | 448 | 3,160 | 0.88 (0.78-1.01) | 416 | 2,552 | 0.96 (0.85-1.08) | 411 | 2,328 | 1.00 (0.89-1.12) |
| Q5 (most privileged) | 70 | 500 | 1.00 (Reference) | 349 | 2,077 | 1.00 (Reference) | 496 | 2,849 | 1.00 (Reference) | 606 | 3,367 | 1.00 (Reference) |

Note: CI = confidence interval; FR = fecundability ratio; PRESTO = Pregnancy Study Online; Adjusted for age, calendar year of enrollment, and geographic region of residence

**eTable 16.** Associations between economic, racial, and racialized economic segregation with fecundability accounting for adjustments in the analytic sample related to residential mobility and geospatial exposure assessment, PRESTO 2013-2022

|  | **Restricted to participants who have the same zip-code within the 12 months before enrollment** | | | **Includes participants with**  **a zip-code level geocode** | | |
| --- | --- | --- | --- | --- | --- | --- |
|  | (n=5,153) | | | (n=11,040) | | |
|  | **Pregnancies** | **Cycles** | **Adjusted FR**  **(95% CI)** | **Pregnancies** | **Cycles** | **Adjusted FR**  **(95% CI)** |
| **ICE_income_** |  |  |  |  |  |  |
| Q1 (most disadvantaged) | 446 | 3,749 | 0.77 (0.69-0.87) | 963 | 8,166 | 0.80 (0.74-0.86) |
| Q2 | 542 | 3,907 | 0.84 (0.76-0.93) | 1,189 | 8,753 | 0.86 (0.80-0.92) |
| Q3 | 659 | 4,444 | 0.89 (0.80-0.98) | 1,349 | 9,359 | 0.89 (0.83-0.96) |
| Q4 | 757 | 4,661 | 0.96 (0.87-1.05) | 1,463 | 9,381 | 0.94 (0.88-1.01) |
| Q5 (most privileged) | 786 | 4,538 | 1.00 (Reference) | 1,588 | 9,450 | 1.00 (Reference) |
| **ICE_white/black_** |  |  |  |  |  |  |
| Q1 (most disadvantaged) | 537 | 3,783 | 0.94 (0.84-1.05) | 1,098 | 8,267 | 0.94 (0.86-1.01) |
| Q2 | 652 | 4,325 | 0.97 (0.87-1.08) | 1,363 | 9,539 | 0.96 (0.89-1.03) |
| Q3 | 686 | 4,620 | 0.97 (0.88-1.07) | 1,392 | 9,452 | 0.99 (0.92-1.06) |
| Q4 | 671 | 4,351 | 1.00 (0.90-1.10) | 1,396 | 9,124 | 1.02 (0.95-1.09) |
| Q5 (most privileged) | 644 | 4,220 | 1.00 (Reference) | 1,303 | 8,727 | 1.00 (Reference) |
| **ICE_income + white/black_** |  |  |  |  |  |  |
| Q1 (most disadvantaged) | 446 | 3,739 | 0.75 (0.67-0.84) | 989 | 8,154 | 0.80 (0.74-0.86) |
| Q2 | 592 | 4,100 | 0.86 (0.77-0.95) | 1,204 | 9,025 | 0.83 (0.77-0.89) |
| Q3 | 601 | 4,149 | 0.84 (0.76-0.93) | 1,264 | 9,089 | 0.85 (0.79-0.91) |
| Q4 | 734 | 4,683 | 0.90 (0.83-0.99) | 1,484 | 9,529 | 0.92 (0.87-0.99) |
| Q5 (most privileged) | 817 | 4,628 | 1.00 (Reference) | 1,611 | 9,312 | 1.00 (Reference) |
| **ICE_education_** |  |  |  |  |  |  |
| Q1 (most disadvantaged) | 435 | 3,423 | 0.81 (0.73-0.91) | 912 | 7,659 | 0.77 (0.72-0.84) |
| Q2 | 525 | 3,975 | 0.82 (0.74-0.91) | 1,133 | 8,722 | 0.80 (0.75-0.86) |
| Q3 | 632 | 4,306 | 0.90 (0.81-0.99) | 1,344 | 9,371 | 0.87 (0.81-0.93) |
| Q4 | 764 | 4,658 | 0.98 (0.90-1.08) | 1,489 | 9,582 | 0.93 (0.88-1.00) |
| Q5 (most privileged) | 834 | 4,937 | 1.00 (Reference) | 1,674 | 9,775 | 1.00 (Reference) |
| **ICE_white/hispanic_** |  |  |  |  |  |  |
| Q1 (most disadvantaged) | 556 | 3,976 | 0.95 (0.85-1.06) | 1,124 | 8,565 | 0.93 (0.86-1.00) |
| Q2 | 647 | 4,093 | 1.05 (0.95-1.17) | 1,356 | 9,300 | 1.00 (0.93-1.08) |
| Q3 | 675 | 4,478 | 1.01 (0.91-1.12) | 1,396 | 9,354 | 1.01 (0.94-1.09) |
| Q4 | 668 | 4,426 | 1.00 (0.90-1.11) | 1,370 | 8,992 | 1.02 (0.95-1.10) |
| Q5 (most privileged) | 644 | 4,326 | 1.00 (Reference) | 1,306 | 8,898 | 1.00 (Reference) |
| **ICE_white/asian_** |  |  |  |  |  |  |
| Q1 (most disadvantaged) | 605 | 4,151 | 0.99 (0.89-1.10) | 1,196 | 8,837 | 0.97 (0.90-1.05) |
| Q2 | 655 | 4,257 | 1.03 (0.93-1.14) | 1,321 | 9,119 | 1.02 (0.94-1.09) |
| Q3 | 650 | 4,449 | 0.99 (0.89-1.09) | 1,384 | 9,427 | 1.02 (0.95-1.09) |
| Q4 | 654 | 4,211 | 1.04 (0.94-1.15) | 1,385 | 8,981 | 1.06 (0.99-1.14) |
| Q5 (most privileged) | 626 | 4,231 | 1.00 (Reference) | 1,266 | 8,745 | 1.00 (Reference) |
| **ICE_income + white/hispanic_** |  |  |  |  |  |  |
| Q1 (most disadvantaged) | 456 | 3,754 | 0.76 (0.68-0.85) | 978 | 8,217 | 0.79 (0.73-0.85) |
| Q2 | 529 | 3,877 | 0.81 (0.73-0.90) | 1,156 | 8,590 | 0.84 (0.78-0.90) |
| Q3 | 674 | 4,578 | 0.86 (0.78-0.94) | 1,358 | 9,498 | 0.87 (0.82-0.93) |
| Q4 | 727 | 4,572 | 0.91 (0.83-1.00) | 1,456 | 9,488 | 0.91 (0.85-0.97) |
| Q5 (most privileged) | 804 | 4,518 | 1.00 (Reference) | 1,604 | 9,316 | 1.00 (Reference) |
| **ICE_income + white/asian_** |  |  |  |  |  |  |
| Q1 (most disadvantaged) | 474 | 3,924 | 0.76 (0.68-0.85) | 1,007 | 8,330 | 0.80 (0.74-0.86) |
| Q2 | 561 | 3,963 | 0.85 (0.76-0.94) | 1,174 | 8,911 | 0.83 (0.77-0.89) |
| Q3 | 616 | 4,234 | 0.84 (0.76-0.93) | 1,274 | 9,093 | 0.85 (0.80-0.91) |
| Q4 | 725 | 4,565 | 0.92 (0.84-1.01) | 1,492 | 9,436 | 0.94 (0.88-1.01) |
| Q5 (most privileged) | 814 | 4,613 | 1.00 (Reference) | 1,605 | 9,339 | 1.00 (Reference) |

Note: CI = confidence interval; FR = fecundability ratio; PRESTO = Pregnancy Study Online; Adjusted for age, calendar year of enrollment, and geographic region of residence

**eTable 17.** Natural direct and indirect effects of racialized economic segregation (ICE_income + white/black_) in the most disadvantaged (Q1) vs. most privileged (Q5) quintile and fecundability, PRESTO 2013-2022

| Mediator | Natural Indirect Effect | Natural Direct Effect | Percentage Mediated, % |
| --- | --- | --- | --- |
|  | Adjusted FR (95% CI) | Adjusted FR (95% CI) |  |
| Irregular cycles (yes vs. no) | 0.98 (0.97-0.99) | 0.80 (0.74-0.87) | 6.7 |
| Infrequent menstrual cycles (>38 days: yes vs. no) | 1.00 (1.00-1.00) | 0.79 (0.73-0.85) | 0.1 |
| Frequent menstrual cycles (<24 days: yes vs. no) | 1.00 (0.98-1.02) | 0.79 (0.72-0.86) | 0.3 |
| Less than 16 years educational attainment (yes vs. no) | 0.95 (0.93-0.97) | 0.83 (0.76-0.90) | 21.0 |
| Current unemployment (yes vs. no) | 1.00 (1.00-1.01) | 0.78 (0.72-0.85) | – |
| Current body mass index (≥30 vs. <30 kg/m^2^) | 0.93 (0.91-0.95) | 0.84 (0.77-0.91) | 25.8 |
| Current smoker (yes vs. no) | 0.99 (0.98-1.00) | 0.80 (0.74-0.87) | 5.1 |
| Current alcohol intake (≥7 vs. <7 drinks/week) | 1.00 (0.99-1.01) | 0.79 (0.72-0.85) | 0.7 |
| Sleep duration (<7 vs. ≥7 hours/night) | 0.99 (0.97-1.00) | 0.80 (0.73-0.86) | 5.1 |
| History of STI (yes vs. no) | 0.98 (0.97-1.00) | 0.80 (0.74-0.87) | 5.9 |
| History of uterine leiomyomata (yes vs. no) | 1.00 (0.99-1.00) | 0.79 (0.73-0.86) | 1.1 |
| History of endometriosis (yes vs. no) | 1.00 (1.00-1.00) | 0.79 (0.73-0.85) | 0.5 |
| History of polycystic ovary syndrome (yes vs. no) | 0.99 (0.98-0.99) | 0.80 (0.73-0.86) | 5.3 |
| High perceived stress (PSS score: ≥25 vs. <25) | 0.99 (0.99-1.00) | 0.79 (0.73-0.86) | 1.8 |
| Severe depressive symptoms (MDI score: ≥30 vs. <30) | 0.99 (0.98-1.00) | 0.79 (0.73-0.86) | 3.9 |

Note: CI = confidence interval; FR = fecundability ratio; MDI = Major Depression Inventory; PRESTO = Pregnancy Study Online; PSS = Perceived Stress Scale; Q = quintile; STI = sexually transmitted infection (defined as chlamydia, genital herpes, or genital warts); Adjusted for age, calendar year of enrollment, and geographic region of residence; Participants with an ICE_income + white/black_ quintile 2-4 are excluded; **Percentage mediated is not reported when the natural indirect and natural direct effects are in opposite directions.**
